# Strategies Implemented by Public Institutions to Approach the Judicialization of Health Care in Brazil: A Systematic Scoping Review

**DOI:** 10.3389/fphar.2020.01128

**Published:** 2020-07-30

**Authors:** Sueli Miyuki Yamauti, Jorge Otavio Maia Barreto, Silvio Barberato-Filho, Luciane Cruz Lopes

**Affiliations:** ^1^ Graduate Program in Pharmaceutical Science, University of Sorocaba, Sorocaba, Brazil; ^2^ Oswaldo Cruz Foundation, Brasília, Brazil

**Keywords:** Brazil, judicialization of health care, organization and administration, public health, lawsuits and litigation, justice administration system, delivery of health care, health systems

## Abstract

**Background:**

The judicialization of health care is a social claim concerning the right to the access to health care. It usually occurs due to gaps in public policy or failures in its application. In Brazil, several public institutions have implemented strategies to approach this phenomenon. However, these strategies have not yet been systematized into functional categories.

**Objective:**

To categorize and analyze the strategies implemented by public institutions in Brazil to approach the judicialization of health care.

**Method:**

A systematic scoping review was developed following the method proposed by the Joanna Briggs Institute. The descriptor ‘judicialization of health’ was used to conduct the searches for studies in 18 electronic databases and other types of documents in the gray literature until March 2019. Documents containing the reports of strategies implemented in public institutions to approach the judicialization of health care in Brazil were included. Two independent reviewers assessed the eligibility of the documents and extracted the data. The strategies identified were categorized using definitions from the World Health Organization and existing Brazilian legislation.

**Results:**

Seventy eight implemented strategies were identified and organized into nine categories: i. Technical support to the judiciary; ii. State health committees; iii. Organization of assistance; iv. Compliance with court orders, v. Computerized information systems; vi. Administrative proceeding; vii. Defense of the public authority; viii. Pharmacy and therapeutics committee; ix. Alternative dispute resolution. These categories are not mutually exclusive and often act in concert or complement each other’s activities. They represent services either existing or provided for in legal provisions by the public administration to meet various types of demands.

**Conclusions:**

The categories proposed to approach the judicialization of health care represent some of the recommendations for qualifying public administration or are provided for in Brazilian legislation, or both. The existence of recommendations and legislation facilitate, but do not guarantee, the implementation of strategies by public institutions.

## Introduction

The judicialization of health care, mainly in Latin America and the Caribbean, can be considered as a social claim to a pressing need: access to adequate health care, whether services or medical supplies, is currently not being met due to various reasons. Among these reasons are the lack of, or inadequacy of, health coverage; limitations or inequality in access to health care; and the poor quality of service delivery (Pinzón-Flórez et al., 2016).

In Brazil, the judicialization of health emerged with the activism of people who live with the HIV virus who demanded drug treatment (Tribunal de Contas da União, 2017). This happened shortly after the promulgation of the Federal Constitution in 1988 and the creation of the Unified Health System (SUS in Portuguese) in 1990, where for the first time, the Brazilian government considered health as a fundamental right of those who live in the country (Brasil, 1988; Brasil, 1990).

SUS is its main health policy and one of the largest social inclusion policies in the world. It provides universal and comprehensive care to all people without distinction in order to guarantee access to health care. Social participation is an important factor for the construction of SUS and for reducing inequities (Vieira et al., 2014; Ministério da Saúde, 2019b).

However, in Brazil, the claim of these rights through judicialization of health care can deepen the inequalities in access to health care (Chieffi and Barata, 2009).

The judicialization, especially when instigation factors are avoidable, has a negative functional and financial impact on both public health care and legal institutions. Their results often bring an increase in the number of services that must be offered, health technology expenditures when health care needs could be more efficiently met using other products that existing in the public health care system, and significant costs associated with lawsuits. Furthermore, it can also lead to fines, seizing of funds, and even imprisonment of managers for noncompliance with court order (Vieira and Zucchi, 2007; Delduque et al., 2013; Tribunal de Contas da União, 2017; Vargas-Pelaez et al., 2019).

The effect of increase in the number of lawsuits is alterations in the health care delivery that public institutions are able to provide to the public, in that these institutions now need to prioritize compliance with court orders to the detriment of systemic rationality in the provision of access to health care (Chieffi and Barata, 2009; Tribunal de Contas da União, 2017; Vargas-Pelaez et al., 2019).

Public Administration driven by pressures to expand services and health care technologies, is being institutionally reorganized to meet these demands (Vargas-Pelaez et al., 2019). However, the strategies used to approach the judicialization in Brazil, in both the legal and health areas, have not yet been categorized as to their organization and operation.

The purpose of this review is to categorize and analyze the institutional strategies used by the public sector to approach the judicialization of health care in Brazil.

## Method

This scoping review used the following guidelines: the Joanna Briggs Institute guidelines for conducting systematic scoping reviews (Levac et al., 2010; Peters et al., 2015); the elaboration of narrative syntheses for systematic reviews produced by Popay et al. (2006); and the Preferred Reporting Items for Systematic reviews and Meta-Analyzes extension for Scoping Reviews (PRISMA-ScR) checklist (Tricco et al., 2018) (**Appendix 1**).

### Eligibility Criteria

#### Inclusion and Exclusion Criteria

Both primary and secondary studies were analyzed, regardless of their study design or publication status. Abstracts and presentations given at scientific events, newsletters and administrative reports, as well as news published on the internet were also scrutinized.

Documents were included concerning any type of institutional strategy that has been implemented to approach the judicialization of health care in Brazil, both in the areas of health care management and legal^1^.

Documents were excluded from the analysis when merely: i. described or characterized the judicialization of health, according to the type of action, the characteristics of the actors involved, number of lawsuits, etc.; ii. analyzed specific lawsuits from the point of view of the plaintiffs’ needs; iii. suggested strategies that were not in fact implemented; iv. cited the name of an implemented strategy without providing sufficient information to characterize it; v. reported strategies that were used by lawyers, the public defender’s office or the public prosecution office to judicialize health care demands.

#### Concepts

Considering the objectives of this review, the following concepts were employed:

Judicialization of health care: A multifaceted phenomenon, present in Latin America and the Caribbean in a similar way, where a lawsuit is filed against an institution of the public health care sector due to gaps in health policy or health care delivery failures (Perlingeiro, 2014). The purpose of the lawsuit is to guarantee the person’s, individually or collectively, constitutional right to health care. The term covers all solicitations related to the provision of health care, including medicines, equipment and procedures, materials, hospitalizations, medical care, care programs, protocols (Vieira, 2016), and any materials deemed necessary for providing health care to the population in order to minimize or resolve diseases or health problems (Panerai and Peña-Mohr, 1989). However, it does not include litigation for medical act or medical malpractice, for rights of the dying or judicialization of end of life medical decision-making, for reproductive rights of women or abortion rights and lawsuits for compulsory hospitalization. In Brazil, the court’s decision is almost always in favor of the plaintiff (Advocacia-Geral da União et al., 2017).

Strategy, regarding health care systems: as defined by the World Health Organization, is “a series of broad lines of action intended to achieve a set of goals and targets set out within a policy or programme” (World Health Organization, 2019). It differs from ‘intervention’, which is defined as “an activity or set of activities aimed at modifying a process, course of action or sequence of events, in order to change one or several of their characteristics such as performance or expected outcome” (World Health Organization, 2019). Although strategy and intervention are distinct activities, in the health policy literature, there is a prevalence of the use of the word strategy, and the studies on the topic generally use ‘strategy’ to the detriment of ‘intervention’.

Therefore, this scoping review adopted the term ‘strategy’ to define any activity, action, service, experience or practice, professional or otherwise, implemented in public institutions in order to approach the judicialization of health care.

#### Context and Institutions Involved

Public sector institutions, both health and legal, that are affected by the judicialization of health care have as a consequence an increase in the demand for services which in turn results in an increased need for public spending (Asensi and Pinheiro, 2015; Advocacia-Geral da União et al., 2017; Conselho Nacional de Justiça, 2017; Schulze, 2017). This can compromise the health care and legal services provided for all users and can also serve to disrupt these institutions, both administratively and financially (Siqueira, 2015; Tribunal de Contas da União, 2017). Consequently, these institutions are required to reorganize and reallocate resources earmarked for other purposes in order to comply with court orders for health care technologies (Tribunal de Contas da União, 2017; Vargas-Pelaez et al., 2019).

To circumvent such situation, several public institutions have created their own strategies to avoid or minimize the judicialization of health care in the provision of public services. However, yet there is no categorization of these strategies that can be used to conduct further studies on the subject.

### Search Strategy

The terms of the Brazilian descriptor “judicialization of health” (judicialização da saúde, in Portuguese) (Descritores em Ciência da Saúde (DeCS), 2017) were used both alone and in combination for this search strategy (**Appendix 2**).

### Information Sources

The search was performed from between data inceptions databases until March 2019, without limits on language. The search was limited to documents from Brazil. The following electronic information bases were used:

1. OneFile, Scopus, Social Sciences Citation Index, Expanded Sciences Citation Index (Web of Science), Directory of Open Access Journals (DOAJ), Sociological Abstracts, MEDLINE/PubMed, Scientific Electronic Library Online (SciELO), SciELO Brazil, JSTOR Archival Journals, NDLTD Union Catalog, SAGE Journals and Publications, Science Direct Journals and Books, Elsevier, Oxford Journals, Materials Science & Engineering Database, Cambridge Journals, and Dialnet. The searches in these databases were carried out *via* the Digital Library of Scientific Journals of the Brazilian Federal Agency for the Improvement of Higher Education (Portal CAPES, in Portuguese)^2^.
2. Gray literature websites: CAPES Thesis Bank and Digital Library of Theses and Dissertations (BDTD) *via* Portal CAPES; Federal Pharmacy Council; Innovare Institute; National School of Public Administration; National Council of Health Secretaries; National Council of Justice; and websites of public agencies promoting awards in order to increase the dissemination of successful practices in the areas of public health, law and administration.

### Other Search Resources

In documents reporting the implementation of strategies, a lack of specifics about the strategies themselves is a fact that was predicted and described by Popay et al., (2006). Therefore, to circumvent this problem, a manual search was performed using the references and citations in the selected documents in order to locate strategies unidentified in the original search and to complement the information of documents with insufficient data for data extraction.

The webpages of courts, municipal or state health departments, attorney offices, public prosecutor’s offices, public defender’s offices, the Brazilian ministry of health, and other institutions that have implemented strategies of interest for this study were also scrutinized.

### Study Selection Process

The selection of titles and abstracts was performed by two independent reviewers (SY and LL) based upon the eligibility criteria. Any disagreements between the reviewers were resolved by consensus.

### Data Extraction

Reviewer’s calibration was performed in screening full documents by extracting at least three articles until consensus was reached. This procedure occurred until the reviewers could extract the data they identified:

–Article (authors, year of publication, title, and type of publication);
–Study (city/state, year of implementation and name of strategy adopted by the institution responsible for its implementation);
–Strategy (objectives, how it functioned in practice and results).

### Categorization of Findings, Analysis and Synthesis of Results

The implemented strategies identified in this scoping review were grouped according to their stated objectives and the similarity of activities developed and described in the included documents. They were categorized according to concepts used by the Pan American Health Organization/World Health Organization regarding existing services or activities in health care (Holloway and Green, 2003; Ribeiro et al., 2004; WHO Regional Office for the Western Pacific, 2004; Health Metrics Network and World Health Organization, 2008). For strategies related to Public Administration or the legal area, definitions based on Brazilian legislation were used (Brasil, 1999; Brasil, 2010; Conselho Nacional de Justiça, 2010a; Conselho Nacional de Justiça, 2010b; Brasil, 2015; Conselho Nacional de Justiça, 2016b).

Explanatory tables and figures were then elaborated about the proposed categories, along with a narrative synthesis about each category’s characteristics.

## Results

### Document Selection and Composition of the Identified Strategies

**Figure 1:**
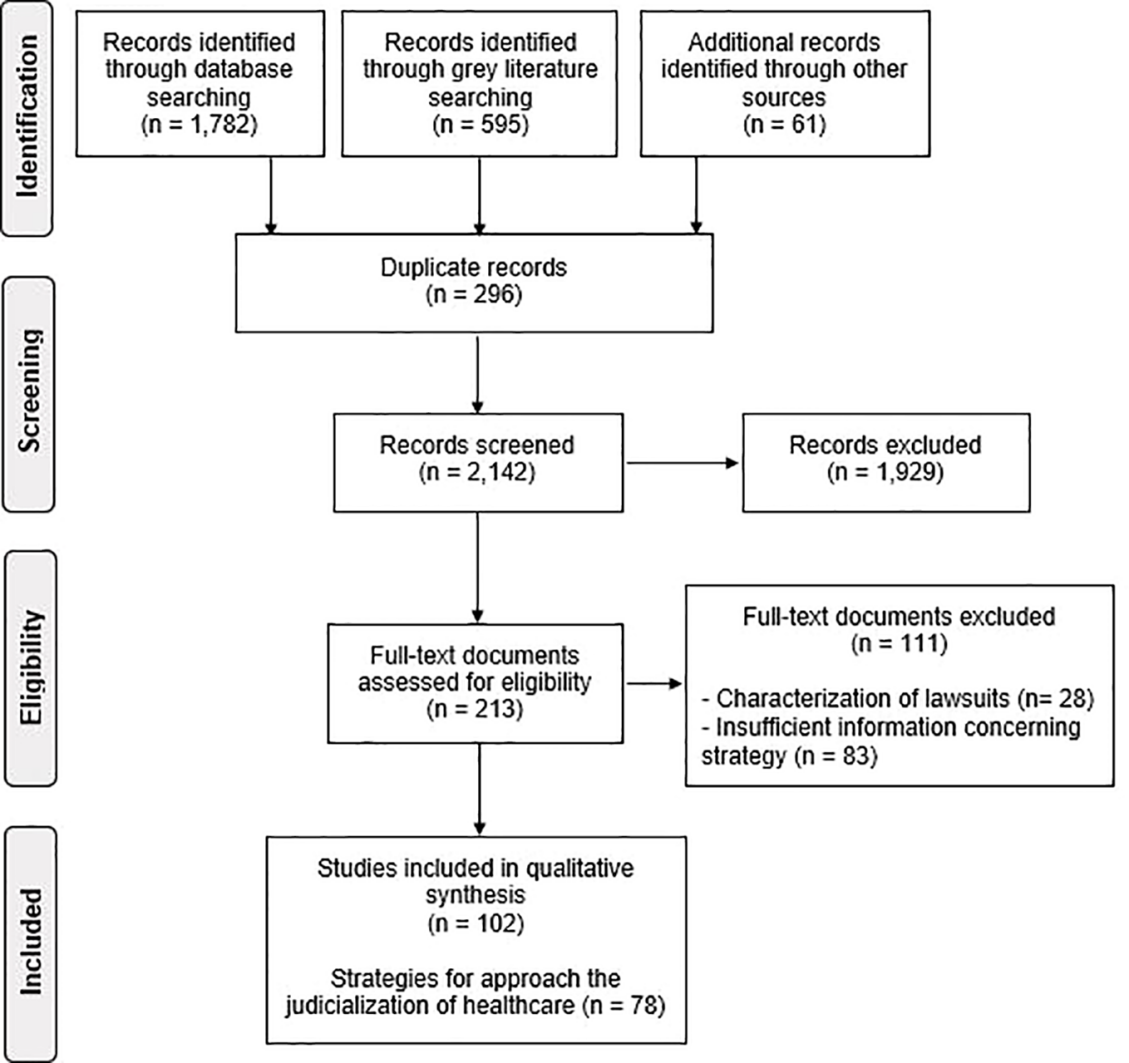
Flow diagram for the scoping review process based on Preferred Reporting Items for Systematic Reviews and Meta-Analyses (PRISMA).

The initial search for this study retrieved 2,377 documents, of which 2,225 documents were removed during the screening of titles and abstracts and 111 documents were excluded after a reading of the full text. Sixty one documents were found by manual search in other search resources. The final selection included 102 documents containing 78 strategies implemented by Brazilian public institutions to approach the health care judicialization (**Figure 1** **)**.

### Categorization of Institutional Strategies

**Figure 2:**
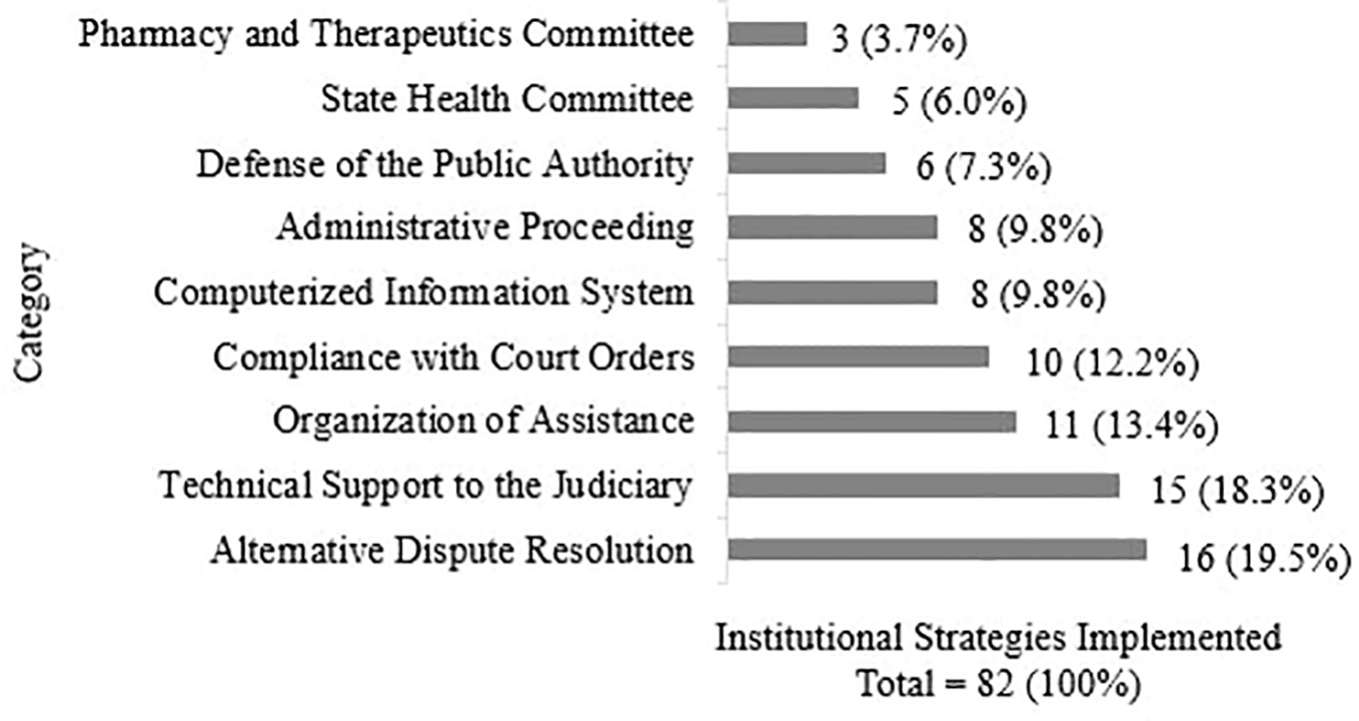
Number of institutional strategies implemented to approach the judicialization of health care by category.

Seventy eight implemented institutional strategies were identified in this review. After being grouped, they resulted in the proposition of nine categories of strategies to approach the judicialization of health care (**Table 1** and **Figure 2** **)**.

**Table 1:** Categorization of institutional strategies implemented to approach the judicialization of health care.

**Category**	**Explanation**
***Legislation***	
**Alternative Dispute Resolution**	The consensual settlement of conflicts is provided for in Law No. 13,105 of March 16, 2015 (WHO Regional Office for the Western Pacific, 2004) of the Brazilian Civil Procedure Code, which decrees in its paragraphs 2 and 3 of article 3 that conciliation, mediation and other methods of consensual dispute resolution should be encouraged by judges, lawyers, public defenders and prosecutors, including during the course of the judicial process, and should also be promoted by the State whenever possible.
**Technical support to the judiciary**	This category encompasses the technical support centers for the judiciary and services that provide technical reports on health care technologies. It is based on the Resolution of the National Council of Justice (CNJ) No. 238 of September 6, 2016 (Health Metrics Network & World Health Organization, 2008), which establishes the creation of Technical Support Centers for the Judiciary,> made up of health professionals, to prepare opinions based upon the best available evidence; and, CNJ Recommendation No. 31 of March 30, 2010 (Ribeiro et al., 2004), which recommends that courts take steps to better support judges and other legal operators to ensure greater efficiency in resolving legal claims involving health care.
**Compliance with court orders**	Category based on Law No. 13,105, of March 16, 2015, of the Civil Procedure Code (WHO Regional Office for the Western Pacific, 2004), explains in its paragraphs 1, 2 and 3 of Article 203 that the pronouncements or declarations of the resolutions taken by the Judge shall consist of: sentence—ends the cognitive phase of the common procedure and extinguishes the execution; interlocutory decision—is any judicial pronouncement of a decisional nature that does not fall as a sentence; or orders—other pronouncements practiced in the process, of office or at the request of the party. These resolutions must be enforced by the judicialized institutions under penalty of fines, seizing of funds or imprisonment of the institutional representative.
**Administrative proceeding**	This category is based on Law No. 9,784, of January 29, 1999 (Conselho Nacional de Justiça, 2016b), which regulates the administrative proceeding within the Federal Public Administration, in its articles 5, 6 and 7, defines that the administrative proceeding must be carried out subsequent to a request from the interested party by means of a letter or standardized form with the following data: I—administrative body or authority to which it is addressed; II—identification of the person concerned or who represents him; III—domicile of the applicant or place to receive communications; IV—formulation of the request, with statement of the facts and their grounds; V—date and signature of the applicant or his representative. The public servant should advise the interested party of any problems and may not unreasonably refuse to receive the documents. The administrative proceeding is also present at the state and municipal levels.
**Defense of the public authority**	Presidential Decree No. 7,392, of December 13, 2010 (Conselho Nacional de Justiça, 2010a), which approves the regimental structure and the organization of commission positions of the Attorney General of the Union (AGU); defines in its annex I that the AGU is the institution that represents and defends the Union, judicially and extra judicially, through legal advice and legal processing in service to the Executive Power. The Federal Attorney General's Office performs such functions over federal autarchies and foundations, the State Attorney General's Office and the Municipal Attorney General's Office act within the State and municipal governments, respectively.
**State Health Committee**	State health committees are provided for in CNJ Resolution No. 238 of September 6, 2016 (Health Metrics Network and World Health Organization, 2008), which provides for the establishment and maintenance of State Health Committees by the Federal Courts and Regional Courts; and, CNJ Resolution 107, of April 6, 2010 (Brasil, 2015), which states that these committees have the same duties as those for the National Executive Committee. It is noteworthy that such committees should propose concrete and normative measures aimed at the prevention of judicial conflicts, define strategies on health law issues and assist the courts in the creation of Judiciary Technical Support Centers. In addition, each committee should have multi-professional representation which, at a minimum, includes of Magistrates, health care managers, and other participants in the Health and Judicial System.
***Recommendations for qualifying health services***	
**Organization of Assistance**	“The organization of care is based on the ordering of health care management, informed by local situations as well as municipality as a whole, responding to the needs of the population, either within the scope of Primary Health Care/Family Health or specialized attention at all levels of resolution” (Siqueira, 2015). This category includes any and all strategies implemented with a primary focus on the planning, organization and management of services provided to the population and the pharmaceutical care cycle (selection, procurement, distribution, storage, dispensing).
**Computerized Information System**	Information and communication technology are a set of instruments consisting of computers, software, data capture devices, wireless communication devices, and local and long-distance networks that move information. It also includes the people who design, implement and support these tools and the information system (Descritores em Ciência da Saúde (DeCS), 2017). An information system is a tool that provides information support for the decision-making process at each level of an organization (Holloway and Green, 2003). In this context, a computerized information system is a system that provides information about the citizen and the provision of services and/or to manage health and/or legal demands using information and communication technology.
**Pharmacy and Therapeutics Committee**	The Pharmacy and Therapeutic Committees (Asensi and Pinheiro, 2015) are forums that bring together everyone involved in drug-use decisions at any level within a healthcare system. Its role is to develop drug policies, in addition to having a technical-advisory character to the health and administrative teams for issues related to the Drug Policy and pharmaceutical assistance. It is responsible for the evaluation and selection of products which compose drug formularies and also for the development of clinical protocols and therapeutic guidelines.

Four strategies were allocated into two categories at the same time: ‘Special Requests Review Board’ (Perin et al., 2008; Boldrin, 2014), ‘Araguaína Municipal Technical Support Center’ (Henrique et al., 2013; Asensi and Pinheiro, 2015; Farias, 2016; CEMAS, 2019), ‘Interinstitutional Center for Health Judicialization’ (Secretaria de Estado da Saúde de Alagoas, 2013; Macêdo et al., 2015) and ‘Pharmaceutical and Nutrition Screening and Guidance Services’ (Núcleo de Comunicação Social, 2017; Reis, 2018; Ministério Público do Estado de São Paulo, 2019).

Lists containing information about the characteristics of the strategies and the documents included in this scoping review can be found in **Appendix 3** and **Appendix 4**.

### Characteristics of the Proposed Categories

#### Alternative Dispute Resolution

The implemented strategies focused on alternative dispute resolution methods are the most documented (19.5%). This is partly due to the fact that there exist laws that encourage their use, regardless of the material nature of the matter (Brasil, 1988; Brasil, 2015).

The 16 strategies that make up this category (Pinheiro, 2010; Reis, 2011; Yoshinaga, 2011; Triagem farmacêutica no juizado especial da fazenda pública de São Paulo, 2013; Tribunal de Justiça do Estado do Pará, 2013; Boldrin, 2014; Orsatto, 2014; Asensi and Pinheiro, 2015; Assis, 2015; Galliez, 2015; Macedo, 2016; Nunes, 2016; Souza, 2016; Júnior, 2017; Núcleo de Comunicação Social, 2017; Sant’Ana, 2017) provide for negotiation or inter-institutional dialogue in order to meet health care demands. They also provide for inter-institutional agreements and allow for action without an appeal to the courts. Five of these strategies can also be implemented when there are current legal actions pending (Advocacia Geral da União, 2009; Pinheiro, 2010; Henrique et al., 2013; Queiroz, 2013; Orsatto, 2014; Tavares et al., 2014; Asensi and Pinheiro, 2015; Guimarães and Palheiro, 2015; Paim et al., 2015; Prefeitura Municipal de Lages, 2015; Vasconcellos, 2015; Farias, 2016; Souza, 2016; Cotrim, 2017; Sant’Ana, 2017; CEMAS, 2019). However, the strategies in this category can only be implemented when the matter at hand does not involve an urgency or an emergency.

The meetings for mediation, conciliation or negotiation take place mainly in legal environments (11/15) (Reis, 2011; Yoshinaga, 2011; Triagem farmacêutica no juizado especial da fazenda pública de São Paulo, 2013; Tribunal de Justiça do Estado do Pará, 2013; Queiroz, 2013; Orsatto, 2014; Assis, 2015; Galliez, 2015; Guimarães and Palheiro, 2015; Macedo, 2016; Ringeisen, 2016) with the physical presence of interested parties. Only one of the strategies conducts mediation or conciliation by electronic means (Galliez, 2015).

The public defender is one of the most representative actors in the implementation of these strategies, with the exception of cases which fall into the specialized areas of ‘Health Mediation’—Belo Horizonte (Assis, 2015) and the ‘Pharmaceutical Screening at the Small Claims Courts’ (Triagem farmacêutica no juizado especial da fazenda pública de São Paulo, 2013; Toma et al., 2017). Of the at least six implementations of Alternative dispute resolution strategies found where the public defender’s office is present, it acts as a mediator (Reis, 2011; Pereira, 2012; Assis, 2015; Galliez, 2015; Secretaria de Estado da Saúde do Estado do Maranhão, 2015; Nunes, 2016; Ringeisen, 2016; Secretaria Estadual de Saúde, 2016; Bittencourt, 2017; Cotrim, 2017; Júnior, 2017; Sant’Ana, 2017; Imprensa da DPE/RN, 2019).

Conflict mediation is a dispute resolution strategy that utilizes negotiation facilitated or instigated by a qualified practitioner, intended to reduce or avoid litigation in general (Conselho Nacional de Justiça, 2016a). This process has recently been adapted to better suit the demands for health care technologies and is currently recommended by the Civil Procedure Code, updated in 2015 (Brasil, 2015). It is also presented as one of the options for approach health care judicialization in a synthesis of evidence for health policy (Ministério da Saúde, 2019a). This measure, when it results in agreements between the stakeholders, reduces the costs and expenses associated with the judicialization of health care (Conselho Nacional de Justiça, 2016a).

Some of the reports argue that inter-institutional dialogue facilitates an out-of-court settlement as well as being a cheap, effective and swift strategy (Asensi and Pinheiro, 2015; Instituto de Ensino e Pesquisa, 2019). According to the reported data, the percentage of claims resolved using alternative dispute resolution methods ranged from 26% (Imprensa da DPE/RN, 2019) to 90% (Yoshinaga, 2011; Henrique et al., 2013; Vasconcellos, 2015), but no evidence was found on exactly which method has the best overall effectiveness in resolving health care conflicts.

#### Technical Support to the Judiciary

Strategies based upon the use of technical support centers in support of the judiciary seem to have arisen from Rio de Janeiro’s experience in 2009 with the ‘Technical Advisory Center’ (Silva, 2012). Subsequently, the National Council of Justice (CNJ, in Portuguese) recommended that such centers be established in all courts which deal with health care demands. The establishment of these centers rapidly expanded across the country in two waves: in 2012, after the CNJ Recommendation No. 31 of March 30, 2010 (Conselho Nacional de Justiça, 2010a), and in 2016, following CNJ Resolution No. 238 of September 6, 2016 (Conselho Nacional de Justiça, 2016b).

Centers that provide such technical support to the judiciary are usually denominated with one of two common names: ‘Technical Health Chamber’ (Costa, 2014; Tribunal de Justiça do Estado de Alagoas, 2016) and ‘Judiciary Technical Support Center (NAT-Jus, in Portuguese)’ (Tribunal de Justiça do Estado do Pará, 2013; Costa, 2014; Barros, 2016; Comitê Estadual das Demandas da Saúde do Rio Grande do Norte, 2016; Farias, 2016; Tribunal de Justiça do Estado da Bahia, 2017a; Tribunal de Justiça do Estado da Bahia, 2017b; Tribunal de Justiça do Estado do Ceará, 2017c; Ascom, 2018; Henrique et al., 2018; Mariano et al., 2018; CEMAS, 2019). However, there are centers with other names (Castro, 2012; Boldrin, 2014) that also provide advice or technical reports on health care technologies to the judiciary (Departamento de Informática, 2015; Duarte, 2017).

The judiciary support services identified in this review were implemented in courts at the state level, with the exception that two of these centers were located within teaching hospitals (Perin et al., 2008; Boldrin, 2014; Duarte, 2017) and one at a municipal health department (CEMAS, 2019).

These centers usually have technical cooperation agreements with the health departments, which provide full-time or part-time health professionals for the purpose of preparing advises or technical reports. There are three exceptions to the above: two of the services studied used internal human resources from the court itself (Departamento de Informática, 2015; Tribunal de Justiça do Estado de Alagoas, 2016), and a technical advisory center was hired by the Minas Gerais State Department of Health to clarify the judges’ doubts (Duarte, 2017).

Most of the strategies in this category involve a multi-professional team, with some centers open during all hours that the court is functioning, while others use an ‘on duty’ or ‘on-call’ scheme (Castro, 2012; Silva, 2012; Costa, 2014; Guimarães and Palheiro, 2015; Souza, 2016; Tribunal de Justiça do Estado de Alagoas, 2016; Tribunal de Justiça do Estado da Bahia, 2017a; Tribunal de Justiça do Estado da Bahia, 2017b; Ascom, 2018).

For this purpose, these centers are equipped with standardized documents and technical reports for use, as well as an electronic communication infrastructure and online systems allowing them to respond to requests within 24 h (regardless of the day of the week) or up to five business days.

Its *modus operandi* resembles the existing rapid response services that are available in some countries, where the request for information is processed and answered in a short time (Haby et al., 2016).

Although there exist comparative studies between different NAT-Jus models (Duarte, 2017) and publications describing their history, activities, and service provision (Arruda, 2017; Mariano et al., 2018), there are few studies that have measured the effectiveness of these efforts (Silva, 2012; Instituto de Ensino e Pesquisa, 2019). In addition, some studies and documents reported poor demand for services offered by NAT-Jus (Silva, 2012; Barros, 2016; Tribunal de Contas da União, 2017; Instituto de Ensino e Pesquisa, 2019).

#### Organization of Assistance

The way health service delivery is organized is a determining factor in access to health services goods and services. For this reason, the organizational structure is the focus of attention of the Pan American Health Organization (Pan American Health Organization, 2017), World Health Organization (World Health Organization, 2010a; World Health Organization, 2010b); and the Brazilian Ministry of Health (Ministério da Saúde, 2009; Ministério da Saúde, 2016a; Ministério da Saúde, 2016b).

In this scoping review, it was found that 13.4% of the strategies had as their main objective the reorganization of the care provided: improving service (Mauad et al., 2009; Tavares et al., 2010; Conselho Federal de Farmácia, 2013; Conselho Federal de Farmácia, 2016; Secretaria da Saúde do Município de Ribeirão Preto, 2017); implementing specialized services (Tavares et al., 2009; Tavares et al., 2010; Ungaro, 2011; Secretaria da Saúde do Município de Ribeirão Preto, 2017; Chayamiti, 2018); investing in training and capacity building (Governo do Estado do Espírito Santo, 2007; Tavares et al., 2009; Tavares et al., 2010; Oliveira, 2018); and/or organizing existing services (Bahia et al., 2005; Pontarolli et al., 2015; Comitê Executivo do Fórum Nacional do Judiciário para Saúde do Conselho Nacional de Justiça, 2015; Simões, 2015; Schulze, 2018).

Reports and data from the National Council of Justice from the State of Espírito Santo indicate that these strategies resulted in more agile and effective procedures, reducing the number of active lawsuits, and resolving the cases of unmet court orders (Oliveira, 2018; Instituto de Ensino e Pesquisa, 2019). In addition, savings have been generated through the rational use of high-cost medications resulting from dose sharing and medical supervision in patients with atorvastatin prescription or indication for atorvastatin use (Tavares et al., 2009; Tavares et al., 2010).

In the other Brazilian states, the implementation of strategies focused on the organization of assistance generated important results, such as: i. the detection and arrest of a group of people who were profiting from the sale of biological medicines through lawsuits against public institutions (Sanchez et al., 2008) by monitoring, evaluating and detecting possible frauds in lawsuits (Ungaro, 2011); ii. the decrease in the number of judicial penalties, imposed on both the State government and individual managers (Araújo, 2014), resulting from the restructuring and reallocation of services and professionals from the technical, legal, administrative and assistance sectors in the same physical space (Assis, 2015); and iii. more efficient communication between the actors involved and faster service delivery, using integrated computer systems (data and information control) to guide the reorganization of health service demand response flows (Bahia et al., 2005; Pontarolli et al., 2015; Comitê Executivo do Fórum Nacional do Judiciário para Saúde do Conselho Nacional de Justiça, 2015; Schulze, 2018).

These results suggest that the institutional organization probably helps minimize the judicialization of health care. However, as of 2015, no strategy specifically focused on the organization of assistance was detected, although at that time several Brazilian municipalities experienced institutional disorganization in their health services, especially in the area of ​​pharmaceutical care (Costa E. A. et al., 2017; Costa K. S. et al., 2017; Gerlack et al., 2017; Leite et al., 2017).

#### Compliance With Court Orders

All 10 of the strategies in this category were implemented by health care departments using the existing human resources already available within the service itself. These departments centralized the control and monitoring of court proceedings, including information about plaintiffs and inventories, and the dispensing of medicines, special diets and pharmaceutical materials. They have set protocols and procedures of attendance, usually performing both the initial screening of judicial process to verify the existence of, and the possibility of referral of the complainant to, an already existing service within Brazilian Unified Health System (SUS, in Portuguese) and verifying the need to prepare a technical report for the defense of the public authority.

There exist other services that also assist with extrajudicial demands (Junior, 2008; Tavares et al., 2010; Secretaria de Estado da Saúde de Alagoas, 2013; Macêdo et al., 2015; Guimarães and Palheiro, 2015) and computerized services (Junior, 2008; Tavares et al., 2010; Garofalo et al., 2012; Conselho Federal de Farmácia, 2013; Tribunal de Justiça de Mato Grosso do Sul, 2013; Guimarães and Palheiro, 2015; Pontarolli et al., 2015; Nantes and Dobashi, 2015; Barros, 2016; Secretaria de Estado de Saúde do Mato Grosso do Sul, 2018).

The reports of these strategies indicate that management planning and the structure and organization of both informational and operational activities have led to a more rapid service (Bahia et al., 2005; Junior, 2008; Secretaria de Estado da Saúde do Paraná, 2008; Tavares et al., 2010; Garofalo et al., 2012; Conselho Federal de Farmácia, 2013; Guimarães and Palheiro, 2015; Pontarolli et al., 2015) and in some cases have decreased the expenses associated with judicialization (Junior, 2008; Tavares et al., 2010; Garofalo et al., 2012; Guimarães and Palheiro, 2015).

Two strategies in this category used financing arrangements in order to comply with budgetary burden of court orders. These strategies, however, proved to be insufficient to meet all demands quickly (Pereira, 2012; Tribunal de Justiça de Mato Grosso do Sul, 2013; Simões, 2015; Secretaria de Estado de Saúde do Mato Grosso do Sul, 2018).

#### Computerized Information Systems

The use of computerized information systems integrated with inventory controls intensified in 2005 (Sanchez et al., 2008; Pontarolli et al., 2015).

Some institutions have chosen to adapt their medication control systems, using the specialized component of pharmaceutical care, in order to use these systems concurrently with their control of court demands: ‘Medication Administration System’ (Comitê Executivo do Fórum Nacional do Judiciário para Saúde do Conselho Nacional de Justiça, 2015; Defensoria Pública do Estado do Rio Grande do Sul, 2019); ‘High Cost Drug Management System’ (Conselho Federal de Farmácia, 2013; Pontarolli et al., 2015); ‘National Pharmaceutical Assistance System (Hórus System)’ (Reis, 2011; Ministério da Saúde, Assistência Farmacêutica, 2013). This practice of adaptation has been shown to be more efficient than the development of in-house controls or systems (Garofalo et al., 2012; Pontarolli et al., 2015).

Other institutions have opted to set up systems to standardize processes, reduce miscommunication, and streamline actions and compliance with court orders. The systems that have been developed, such as ‘Coordination of SUS Strategic Demands System’ (Naffah-Filho et al., 2010; Siqueira et al., 2018) and ‘National Register of Opinions, Notes and Technical Information (e-NAT-Jus, in Portuguese)’ (Secretaria-Geral e Departamento de Gestão Estratégica, 2017) contain records of legal opinions and technical information concerning various health technologies. This information is available to the registered user. The ‘Legal Control System’ (Sanchez et al., 2008; Yoshinaga, 2011), ‘Coordination of SUS Strategic Demands System’ (Naffah-Filho et al., 2010; Siqueira et al., 2018) and the ‘Health Chamber System’ (Guimarães and Palheiro, 2015) store information pertinent to court proceedings, such as the plaintiff’s data, dispensations made, tracking information for the entire process and the monitoring of judicial process.

Through the ‘Electronic Portal of Judicial Subpoena” or ‘Online Judicial Order’ (Tribunal de Justiça do Estado do Espírito Santo, 2018; Secretaria de Estado da Saúde do Espírito Santo, 2018), the judiciary electronically forwards any and all documents related to public health care actions in order to promote compliance by the health department. The ‘Extrajudicial Health Procedure’ (Bittencourt, 2017) is an online system used for health care mediation, technical clarifications, and administrative analysis of extrajudicial requests.

Computerized information technologies are now indispensable tools in institutional management, as they simplify many administrative tasks, help control inventories, track user data along with their consumption history, facilitate communication, provide evidence, support decision-making, and aid in the rationalization of available resources (Conselho Nacional de Secretários de Saúde, 2011). These technologies are both recommended and incentivized by the World Health Organization (WHO Regional Office for the Western Pacific, 2004; Health Metrics Network and World Health Organization, 2008) and the Ministry of Health (Brasil, 2016).

Both the Ministry of Health and the National Council of Justice provide computerized information systems free of charge to interested institutions: ‘Coordination of SUS Strategic Demands System’ (Siqueira et al., 2018) and ‘Hórus system’ (Ministério da Saúde, Assistência Farmacêutica, 2013), and ‘e-NAT-Jus’ (Secretaria-Geral e Departamento de Gestão Estratégica, 2017), respectively. Other control systems may also be obtained in the same way, upon request and contract with the health departments (Guimarães and Palheiro, 2015; Pontarolli et al., 2015).

#### Administrative Proceeding

In the strategies involving an Administrative proceeding, the service provision is performed upon request using a standardized form, along with a prescription, medical report and complementary exams. The request can be registered in a computerized system or as a hard copy, and the applicant receives a protocol document for tracking the process. The request may be evaluated by a committee of experts, or merely analyzed as to the feasibility of the service. The response time can vary from 24 h to 45 days (Teixeira, 2011; Yoshinaga, 2011; Conti, 2013; Henrique et al., 2013; Secretaria de Estado da Saúde de Alagoas, 2013; Asensi and Pinheiro, 2015; Guimarães and Palheiro, 2015; Macêdo et al., 2015; Toma et al., 2015; Farias, 2016; Souza, 2016; Núcleo de Comunicação Social, 2017; Secretaria da Saúde do Estado do Amapá, 2017; Toma et al., 2017; Reis, 2018; CEMAS, 2019; Ministério Público do Estado de São Paulo, 2019; Secretaria de Estado da Saúde do Estado de São Paulo, 2019).

Typically, health care services also provide information and clarification on access to medicines within the SUS, and requests for products or services on the SUS funding list are forwarded to the individual units responsible for that specific type of care. They are managed by the health departments, except the ‘Judicial Demand Response Center’ (Teixeira, 2011) which is under the responsibility of the Public Defender’s office.

Addressing health care demands through Administrative proceedings enables the detection of failures in access to SUS and health care technologies which should be evaluated for possible incorporation into SUS. This strategy also helps identify legitimate and just demands for health care technologies, where the individual has exhausted all the existing therapeutic possibilities within SUS to treat their disease or injury (Siqueira, 2015).

The reports of these strategies show that there have been annual increases in health care technology request as well as increases in administrative resolutions (Yoshinaga, 2011; Conti, 2013; Macêdo et al., 2015). All the strategies in this category have reported a decreasing number of health care disputes. However, these data should be analyzed with caution, as what was calculated was based upon the number of administrative proceeding that actually turned into legal proceedings, so it is not possible to know from these reports if all those who applied for administrative proceeding would file a lawsuit if the strategy did not exist.

Thus, there is a need for studies that analyze whether this strategy has actually reduced the number of health care disputes and reduced inequalities in access. Studies are also needed to determine if these strategies are cost-effective and rational, because in Administrative proceedings, it is the manager who determines the items to be offered by the institution, and the manager’s decision is partially based upon purchase volume to decrease per-unit costs (Siqueira, 2015) and is not necessarily based upon the effectiveness of a product (Santos-Pinto, 2013).

#### Defense of the Public Authority

When a public health institution is the defendant in a lawsuit, it is defended in court by attorneys general (Brasil, 2010; Advocacia-Geral da União, 2012), supported by the associated services which provide the necessary technical information (Bandeira et al., 2009; Yoshinaga, 2011; Pereira, 2012).

According to the reports of the strategies in this category, the specialization of attorneys general in health or health law, or of health professionals in the area of law, has generated speed and competence in the defense of the public authority (Sanchez et al., 2008; Yoshinaga, 2011; Pontarolli et al., 2015; Secretaria de Estado da Saúde do Estado de Alagoas, 2015; Toma et al., 2017; Faglioni and Castelo, 2018).

State attorneys who are specialized in health law and who act directly in defense of the state department of health were identified the municipalities of Curitiba/PR, São Paulo/SP and Maceió/AL (Sanchez et al., 2008; Yoshinaga, 2011; Pontarolli et al., 2015; Secretaria de Estado da Saúde do Estado de Alagoas, 2015; Toma et al., 2017; Faglioni and Castelo, 2018). In addition, some reports identified others who perform the administrative function of evaluating public contracts and tenders (Secretaria de Estado da Saúde do Estado de Alagoas, 2015). State prosecutors have entered into partnerships with health secretariats to form integrated teams, supplemented by the use of technical reports prepared by the technical health areas (Sanchez et al., 2008; Yoshinaga, 2011; Secretaria de Estado da Saúde do Estado de Alagoas, 2015; Toma et al., 2017).

These attorney offices also have implemented computerized, standardized and less bureaucratic procedures (Sanchez et al., 2008; Yoshinaga, 2011; Pontarolli et al., 2015; Toma et al., 2017; Faglioni and Castelo, 2018) which facilitate the detection of fraud involving the judicialization of health care (Ungaro, 2011) and the identification of lawsuits where state defense is most likely to succeed (Pontarolli et al., 2015; Faglioni and Castelo, 2018).

Like state prosecutors, those in technical health areas have also sought to specialize in the legal arena, in order to issue better-prepared reports and technical advice in a language accessible to magistrates and prosecutors. Three support services that issue this type of document were detected and all have at least 10 years of existence: ‘Technical Advisory Center of Minas Gerais State Department of Health’ (Pereira, 2012; Pereira and Carneiro, 2012), ‘Technical Report prepared by the Coordination of SUS Strategic Demands’ (Sanchez et al., 2008; Naffah-Filho et al., 2010; Yoshinaga, 2011; Siqueira et al., 2018), and ‘Technical Support Center in Health for the Attorney Office’ (Bandeira et al., 2009; Costa, 2019).

These services are located in health departments and have standardized routines and procedures. They have multi-professional teams including doctors and pharmacists, and work in conjunction with the attorney office. They also use computerized and integrated systems that manage both inventories and lawsuits, helping to avoid duplicate care among health care institutions (Sanchez et al., 2008; Bandeira et al., 2009; Naffah-Filho et al., 2010; Yoshinaga, 2011; Pereira, 2012; Pereira and Carneiro, 2012; Siqueira et al., 2018; Costa, 2019).

Such measures have reduced bureaucracy, streamlined responses to judicial demands, and virtually extinguished punitive measures against the health department due to judicial noncompliance (Sanchez et al., 2008; Bandeira et al., 2009; Naffah-Filho et al., 2010; Yoshinaga, 2011; Costa, 2019). One exception is in the State of Minas Gerais, where managerial, structural and behavioral problems increased the number of health claims and needed to be resolved separately (Pereira, 2012).

#### State Health Committee

The Health Forum, established by Resolution CNJ No. 107/2010 and coordinated by the National Executive Committee, aim to monitor and resolve demands for health care technologies (Conselho Nacional de Justiça, 2010b). The National Committee is composed of the State health committees belonging to the Courts of Justice of the individual States.

Of the 27 State Committees allotted for by legislation, only seven committees are listed on the CNJ website^3^. Sant’Ana (2017) identified six acting committees, three of which differ from those listed on the CNJ website (Sant’Ana, 2017).

However, this scoping review found data sufficient to characterize only five of them: Rio Grande do Sul (Comitê Executivo do Fórum Nacional do Judiciário para Saúde do Conselho Nacional de Justiça, 2015; Schulze, 2018), Paraná (Pontarolli et al., 2015), Tocantins (Farias, 2016; Farias et al., 2016; CEMAS, 2019), Mato Grosso do Sul (Tribunal de Justiça de Mato Grosso do Sul, 2013; Nantes and Dobashi, 2015) and Maranhão (Diniz, 2015; Tribunal de Justiça do Maranhão, 2018).

These committees, composed of at least 12 members, make inter-institutional agreements and are multi-professional, with representatives from the areas of health care, law and the public at large. Except for the Maranhão Committee, the reports indicate that they also promote educational lectures and training to their members, and issue statements and recommendations to the legal area. Only three committees hold monthly meetings, regional meetings and offer training (Tribunal de Justiça de Mato Grosso do Sul, 2013; Pontarolli et al., 2015; Comitê Executivo do Fórum Nacional do Judiciário para Saúde do Conselho Nacional de Justiça, 2015; Nantes and Dobashi, 2015; Schulze, 2018).

Three committees have reports on their activities: i. Rio Grande do Sul, which implemented systemic planning and management actions in order to organize health care in the state. Through these actions, Rio Grande do Sul was able to reduce the number of both new and active judicial actions, as well as to reduce the amount of funds blocked by the judiciary (Schulze, 2018); ii. Mato Grosso do Sul encouraged the implementation of specialized health services and the production of clinical protocols and discovered that meetings and regional training programs produced better results than other strategies used (Tribunal de Justiça de Mato Grosso do Sul, 2013); iii. According to Diniz (2015), between 2012 and 2015 the committee in Maranhão was without human resources to manage it and did not generate any approximation between the Judiciary and the Executive Powers, to the point that most of the magistrates of the state were unaware of the committee’s duties (Diniz, 2015).

The State health committees, when they have active members, appear to improve communication among the various stakeholders involved in health care judicialization, in addition to monitoring and qualifying health services (Tribunal de Justiça de Mato Grosso do Sul, 2013; Pontarolli et al., 2015; Comitê Executivo do Fórum Nacional do Judiciário para Saúde do Conselho Nacional de Justiça, 2015; Nantes and Dobashi, 2015; Farias, 2016; Farias et al., 2016; Schulze, 2018; CEMAS, 2019).

#### Pharmacy and Therapeutic Committee

The use of Pharmacy and therapeutics committees is a strategy that has been recommended by the World Health Organization (Holloway and Green, 2003) since 2003. These committees exist in hospitals, in various municipalities, and at the state and the federal level in Brazil.

The Pharmacy and therapeutics committee is a support body directly linked to the management board of health care institutions. It is responsible for the elaboration and updating of reigning protocols and therapeutic guidelines for clinical practice within the institution, and for the selection, standardization and incorporation of health care technologies and medication. It is made up of a multi-professional team and avails itself to the expertise of *ad hoc* consultants for specific or specialized topics. It has regular meetings at the health departments headquarters and takes an evidence-based approach to the assessment of health care technology (Tavares et al., 2009; Tavares et al., 2010; Secretaria da Saúde do Estado de São Paulo, 2012; Toma et al., 2015; Simabuku et al., 2015; Secretaria de Estado da Saúde do Estado de São Paulo, 2019).

In addition to these activities, the committees also prepare technical reports by legal (Simabuku et al., 2015) and health professionals and provide information on health care technologies upon request, *via* e-mail or official letter. This service can be available to the public (Secretaria da Saúde do Estado de São Paulo, 2012; Toma et al., 2015; Secretaria de Estado da Saúde do Estado de São Paulo, 2019), but in some cases is available only to the pharmacy responsible for dispensing specialty medication (Tavares et al., 2009; Tavares et al., 2010).

These technical reports are provided for the clarification of the author, as well as for the defense of the public power, either as an extrajudicial strategy or in the course of judicial proceedings.

Some reports indicate that the judicialization of health care has resulted in the incorporation of new technology and in the reduction of judicial processes related to such incorporation (Tavares et al., 2009; Tavares et al., 2010; Yoshinaga, 2011; Simabuku et al., 2015), a fact also found in the study by Messeder et al. (2005). However, in Brazil the incorporation of new technologies is still a slow process and with a limited impact on the judicialization of health care (Siqueira, 2015).

### Interrelationship and Interdependence Between Categories

Some categories have activities in common with other categories, such as: extrajudicial (prevention of judicial conflicts) and/or judicial (action based on the filing of the lawsuit); production and provision of technical information to assist in judging health care technology applications; provision of health care products, namely medicines, pharmaceuticals and diets; health care management (service monitoring, standardization of health care technologies, improvements in infrastructure, health care delivery and computerization of services) (**Table 2** **)**.

**Table 2:** Characteristics of categories of institutional strategies implemented to approach the judicialization of health care in Brazil.

Category	Created and implemented according to legislation	Extrajudicial approach	Judicial approach	Production and supply of technical information	Health Products Supply	Health Care Management
**Technical support to the judiciary**	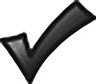		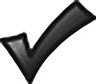	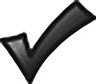	
**Alternative dispute resolution**	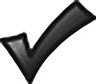	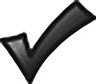	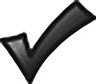		
**Administrative proceeding**	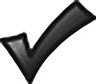	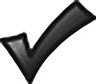			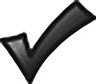	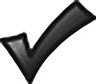
**Compliance with court orders**	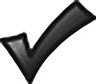		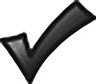		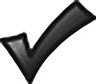	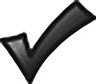
**State health committee**	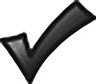	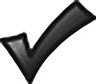				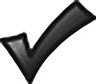
**Defense of the public sector**	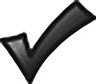		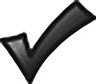	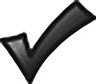		
**Pharmacy and therapeutics committee**		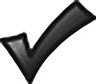	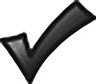	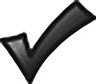		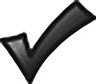
**Organization of assistance**		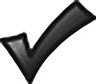	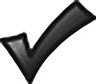		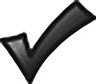	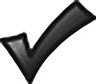
**Computerized information system**		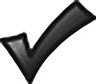	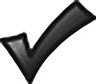	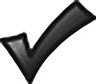		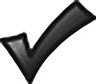

It was observed that there are strategies which involve, in a harmonious and shared manner, services originating from different sectors working in a single physical space, such as the ‘Health Dispute Resolution Chamber’ (Tavares et al., 2014; Guimarães and Palheiro, 2015; Souza, 2016; Sant’Ana, 2017) and the ‘Judicialization of Health Care Assistance Center’ (Assis, 2015; Simões, 2015), in order to reduce bureaucracy and provide faster and more resolute services.

**Figure 3:**
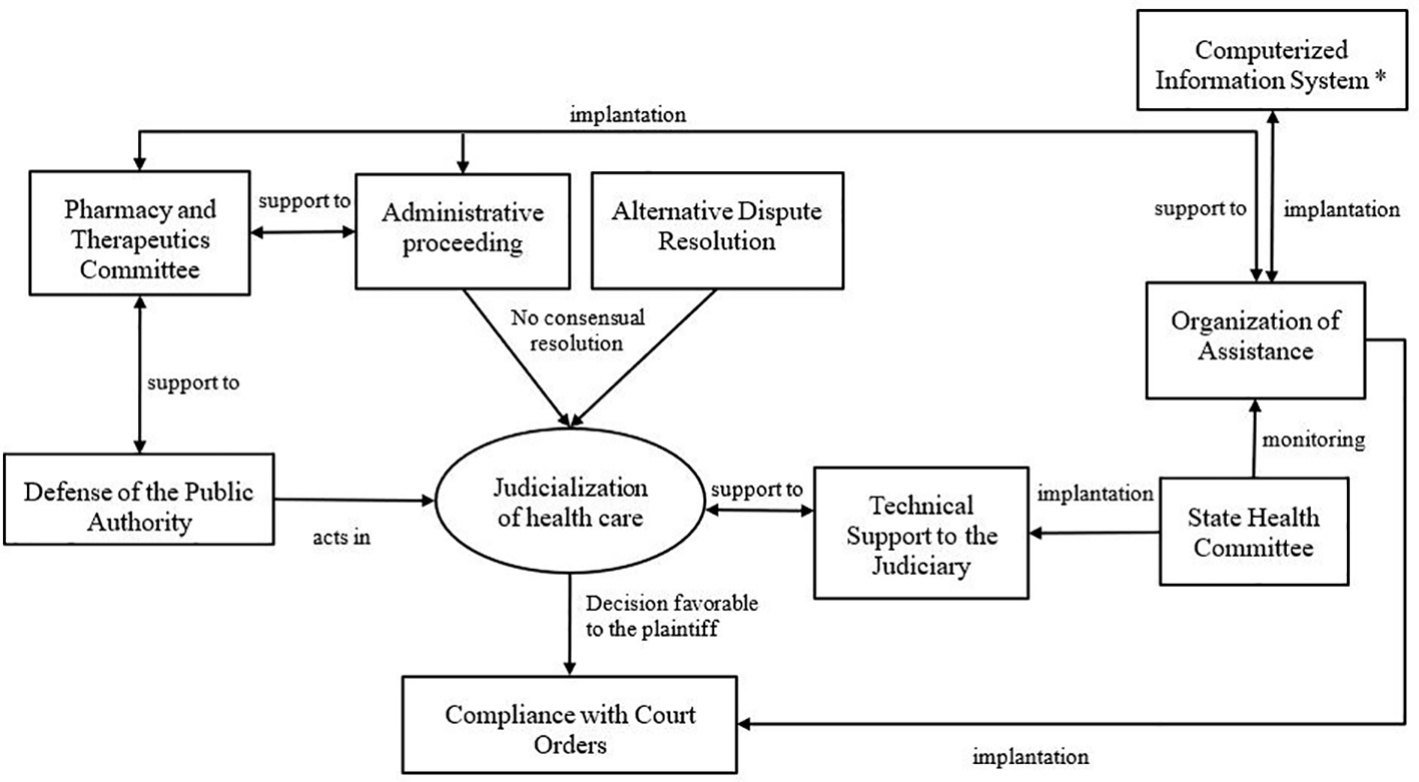
Simplified schematic of the interrelationships existing among categories of strategies for approach the judicialization of health care. *The computerized information system can provide support for all categories of strategies.

A deeper analysis of the categorized strategies allows us to verify activities interrelate between some of them: i. complementary activities: the provision of technical advice, provided by the Pharmacy and therapeutics committee, to the services that act with Administrative proceeding and defense of the public authority; ii. services resulting from a cause–effect relationship: compliance with court decisions favorable to the plaintiff due to the complainant’s rejection of the proposals resulting from Administrative proceeding and/or Alternative dispute resolution methods; iii. synergistic strategies: use of computerized information systems across all categories, whether shared or not, as a management tool (**Figure 3** **)**.

## Discussion

### Main Findings

The 78 institutional strategies implemented to approach the judicialization of health care in Brazil were divided into nine categories with extrajudicial or judicial action, or both, interrelated in a complementary or synergistic manner.

The proposed categories correspond to the activities provided for in both legislation (Brasil, 1999; Brasil, 2010; Conselho Nacional de Justiça, 2010a; Conselho Nacional de Justiça, 2010b; Brasil, 2015; Conselho Nacional de Justiça, 2016b) and in recommendations to improve health care services (Holloway and Green, 2003; World Bank, 2007; Health Metrics Network and World Health Organization, 2008; Ministério da Saúde, 2009; Brasil, 2016; Ministério da Saúde, 2016b). The application of these legal provisions and recommendations are not restricted to a specific area of activity covered, in that any manager can, regardless of their sector of involvement (legal or health), appeal to these provisions.

It was observed that the categories which represent the prevention of conflict appear to be balanced by those categories that represent actions taken after the process of judicialization; the categories of Alternative dispute resolution, Technical support to the judiciary, Organization of assistance and Compliance with court order are those with the greatest number of strategies implemented.

The findings of this review show that public institutions have only implemented those strategies governed within the public management policies and guidelines (Ministério do Planejamento Orçamento e Gestão, Secretaria de Gestão Pública, 2007; Ministério do Planejamento Orçamento e Gestão, Secretaria de Gestão Pública, 2014). This is because the failure of a public administration manager to comply with these precepts is considered as administrative misconduct (Brasil, 1992).

### The Relationship Between the Categories of Strategies With the Feasibility of Legal and Health Systems

It was found that the strategies implemented in public institutions to approach the judicialization of health care in Brazil are strongly linked to the existence of national legal provisions that recommend their implementation and provide them with legal support.

The legal framework is an important factor that facilitates the implementation of these strategies, because it allows the existing services of the public sector to meet various types of demands while permitting that these services be expanded to approach the judicialization of health care, as verified by the strategies in the categories of Administrative proceeding, Defense of the public authority and Alternative dispute resolution.

The findings of this scoping review indicate that, although Brazil is not yet a member of the Organisation for Economic Co-operation and Development (OECD), the Brazilian Public Administration is adopting several OECD recommendations to improve the quality of public service delivery (Organisation for Economic Co-operation and Development, 2018) in order to align with international policies. As well as the Brazilian legal area that is adapting to World Bank recommendations to improve judicial operations in the court system (World Bank, 2004).

These actions can contribute to minimize or solve the existing problems in Public Administration, evidenced by the judicialization of health care. This includes the citizen’s difficulty in accessing goods and health care due to health technologies that do not exist or are not available in the health system, delays in the time required to serve the user, excessive bureaucracy and inefficient communication; the need to generate information and control judicial demands to avoid fraud and duplication of service.

However, not all institutions appear to be able to implement strategies stemming from legal norms, such as NAT-Jus and the State health committee, as there are likely to be unidentified barriers that prevent their implementation, despite the threat of legal enforcement.

In the area of health care, the alignment of the categories with the recommendations concerning adequacy and best practices in public management (Ministério do Planejamento Orçamento e Gestão, Secretaria de Gestão Pública, 2014) and health care systems (World Health Organization, 2010a; Pan American Health Organization, 2017), elaborated by Pan American Health Organization (Pan American Health Organization, 2017), World Health Organization (World Health Organization, 2010a; World Health Organization, 2010b), Ministry of Health (Ministério da Saúde, 2009; Ministério da Saúde, 2016a; Ministério da Saúde, 2016b) and the National Council of State Health Secretaries (Conselho Nacional de Secretários de Saúde, 2011), is useful in assisting in the implementation of strategies focused on the structure of services and the improvement of human resources.

Although strategies in the categories of the Organization of assistance and Computerized information system originate from the recommendations of health institutions, they have universal characteristics and can be used in any field.

The categories of strategies include a combination of strategies and activities implemented simultaneously to approach the judicialization of health care. This is because public institutions are integrated in the same socio-cultural, legal and political context, where one action affects the other (Handtke et al., 2019) inside and outside the institution.

The simultaneous implementation of a series of practices to organize and improve the provision of services are international recommendations (World Bank, 2004; Organisation for Economic Co-operation and Development, 2018), as one activity acts to complement the other and the change in organizational culture must occur throughout the institution.

Thus, it is possible to affirm that, in order to approach the judicialization of health care, systemic planning is necessary in order to both evaluate the vulnerable aspects in Public Administration that should be prioritized for the implementation of strategies, and identify which strategies are most likely to succeed within the local context.

Otherwise, strategies that are destined to fail may be implemented due to lack of supporting services, a fact which could have been identified if a more systematic perspective had been taken. For example, defense of the public sector has a poor chance of being successful if there are no support services to provide the necessary technical information. Likewise, the implementation of services using costly technologies may prove impossible if efficient inventory control is not present.

### Strengths and Limitations of the Study

This study is the first to categorize, describe and analyze feasible and effectively implemented strategies in public institutions to approach the judicialization of health care in Brazil. The proposed categories are based on Brazilian legislation and both international and national recommendations for improving health services.

The Brazilian legislation used in this scoping review is in line with the OECD recommendations on the performance of public services (Organisation for Economic Co-operation and Development, 2018) and the World Bank on the performance of the courts system (World Bank, 2004). It is believed that the alignment of the strategies implemented in Brazil with the recommendations of the OECD and the World Bank facilitates and improves their understanding by various stakeholders.

Although this study used international recommendations strongly based on the best institutional practices, there was little data available on the effectiveness of the strategies implemented, therefore, it was not possible to analyze the impact of these strategies on the Brazilian judicial and health systems.

In addition, it was not possible to compare the strategies implemented in Brazil with those of other countries, as no comparable strategies were found because the subject adopted in this scoping review is rarely published in indexed journals.

Despite these limitations, this scoping review contributes to the systematization of knowledge by facilitating its incorporation into actual management practice and enabling decision makers to properly assess the implementation of one, several or all strategy categories.

Thus, it is expected that countries with governmental systems and with judicialization of health care like Brazil, especially those belonging to Latin America and the Caribbean, can benefit from the proposed categorization.

It is known that the number of strategies presented in this review does not represent all strategies that have been implemented; many strategies encountered did not meet the criteria of inclusion due to the incompleteness of data concerning these strategies. However, it is unlikely that there are institutional strategies which do not fit in one of the proposed categories, as Public Administration is governed by the same principles and guidelines contemplated in the proposed categories, and any variance from these principles and guidelines would be construed as administrative misconduct.

Finally, some of the implemented strategies identified names, given by the institution, that did not indicate the actual activities performed. In these cases, the objectives and various supporting documents were analyzed in order to categorize them.

## Conclusions

The nine categories of strategies proposed to approach health care judicialization interrelate in a complementary or synergistic way. These act both in the prevention of health care conflicts and after the filing of a lawsuit. These also can be used by any type of institution, regardless of the area of expertise.

The existence of legal provisions and recommendations facilitate the implementation of these strategies and services in public institutions, as they guide their structuring and execution. However, this legal support does not guarantee either implementation or success.

### Implications for Public Officials

It is likely that managers in the areas of health care and law already have notions about some of the categories proposed in this review, as some of them are services that originally were used in Public Administration to meet various types of demands and only later were adapted to approach the judicialization of health care.

In addition, public officials interested in implementing such strategies should identify the areas where institutional management is most vulnerable; these areas may prove to be among the main causes of health disputes. Public officials should also analyze whether there is sufficient infrastructure to implement a given strategy.

The consideration of the above aspects may facilitate the choice, or prioritization, of feasible strategies to be implemented in the local context.

### Implications for Researchers

There is a need for studies that better and more fully describe the strategies identified in this scoping review and that are able to assess their respective effectiveness.

The proposed categorization may be useful for further research on the strategies implemented in public institutions to approach the judicialization of health care.

Finally, the development of systematic reviews or policy briefs specific to each of the proposed categories may provide managers with important evidence and help identify those strategies which are most likely to succeed given the local context.

## Data Availability Statement

All datasets generated for this study are included in the article/**Supplementary Material**.

## Author Contributions

SY, JB, and LL contributed conception and design of the study. SY and LL planned the study, performed data extraction and analysis. SY wrote the first draft of the manuscript. SY, JB, SB-F, and LL wrote sections of the manuscript. All authors contributed to the article and approved the submitted version.

## Funding

SY is supported by CAPES Scholarship. The funding source had no part in choosing the topic, in the systematic scoping review or in the decision to submit the article for publication.

## Conflict of Interest

The authors declare that the research was conducted in the absence of any commercial or financial relationships that could be construed as a potential conflict of interest.

## Footnotes

1 In Brazil, the legal area or the justice system is composed primarily of judges and courts of law belonging to the Judiciary Power which are responsible for judging lawsuits, and the essential functions of justice which are composed of the Public Prosecution Office, Public Defender’s Office and Attorney Office which are responsible for the defense of public patrimony, the most vulnerable citizens and the public institutions, respectively. (In: Campos AG. Sistema de justiça no Brasil: problemas de equidade e efetividade. Brasília: IPEA; 2008.).

2 Portal CAPES has free access to journals and databases from different areas of knowledge, acts as a meta-researcher and is available in http://www.periodicos.capes.gov.br/.

3 Available form: http://www.cnj.jus.br/programas-e-acoes/forum-da-saude/iniciativas-dos-comites-estaduais
