# Supplementary material for: Strategies Implemented by Public Institutions to Approach the Judicialization of Health Care in Brazil: A Systematic Scoping Review
Source: Front Pharmacol. 2020 Jul 30;11:1128. doi: 10.3389/fphar.2020.01128 (PMC7406659; doi:10.3389/fphar.2020.01128)
Supplement: Supplementary file 2 [file DataSheet_2.pdf]

frontiers

Supplementary Material

Appendix 2. Database search strategy

1. Digital Library of Scientific Journals of the Brazilian Federal Agency for the Improvement of Higher Education, CAPES Theses Database and Digital Library of Theses and Dissertations of Brazil

1. [Health OR salud OR saúde]
2. AND
3. [Judicialisation OR judicialization OR judicialización OR judicialização]

2. Gray literature

1. Saúde
2. AND
3. [Judicialização OR judiciais OR judicial]
