# Supplementary material for: Strategies Implemented by Public Institutions to Approach the Judicialization of Health Care in Brazil: A Systematic Scoping Review
Source: Front Pharmacol. 2020 Jul 30;11:1128. doi: 10.3389/fphar.2020.01128 (PMC7406659; doi:10.3389/fphar.2020.01128)
Supplement: Supplementary file 3 [file DataSheet_3.pdf]

Supplementary Material

Appendix 3. Identification and categorization of strategies included in the scoping review**.**

No.	County/ state	Year of implementation	Strategy name	Objetives	Strategy Category
1	Ribeirão Preto/SP	2002	Special Requests Review Board [1, 2]	Provide technical advice regarding the request for medicines and equipment from the State Public Prosecution Service (Prosecutor for the Defense of Citizens' Rights of Ribeirão Preto) and the Judiciary of Ribeirão Preto/SP.	Technical support to the judiciary/ Alternative Dispute Resolution
2	Ribeirão Preto/SP	2002	Home visit by the Home Care Service (SAD) (expanded functions) [3, 4]	Promote and protect family health, based on the assistance of an index case, guide the family / caregiver, encourage the development of family / caregiver responsibility for self-care and establish integration mechanism between the health services network and the family.	Organization of Assistance
3	Campo Grande/MS	2005	Lawsuit Coordination [5]	Work on monitoring, advising and complying with lawsuits, as well as streamline and optimize care.	Compliance with court orders
4	Curitiba/PR	2005	Flow of compliance with court order for drug supply [6-9]	Guarantee the population's access to medicines from the programs offered by the Ministry of Health and the Paraná State Department of Health. Monitor and assist compliance with national drug and pharmaceutical assistance policies in the state. Manage the medicines demanded in court.	Compliance with court orders
5	São Paulo/SP	2005	Legal Control System (SCJ) [10, 11]	Concentrate the relevant data from all actions filed (patient name, personal data, disease, medications and products requested, case data, attorney, physician and health service data).	Computerized Information System
6	Vitória/ES	2006	State Commission for Pharmacology and Therapeutics (CEFT) [12, 13]	Periodically update the list of REMEME medicines, participate in the elaboration of clinical protocols for special situations not covered by the Protocols of the Ministry of Health and evaluate requests for non-standard medicines, originated from pharmacies for dispensing exceptional medicines	Pharmacy and Therapeutic Committee
7	Belo Horizonte/ MG	2007	SES MG Technical Advisory Center (NAT / SES MG or Technical Advisory) [14, 15]	Monitor, attend and analyze the lawsuits supporting the State General Counsel, as well as meet extraordinary demands, as established by the Secretary of State for Health.	Defense of the public authority
8	Curitiba/PR	2007	Operating Plan [7, 8]	Provide medications in compliance with court orders, meeting all the requirements of each case individually; adopt virtual flow to optimize the	Organization of Assistance

No.	County/ state	Year of implementation	Strategy name	Objetives	Strategy Category
				time and work processes carried out by the Judicial Demand Drugs Sector, substantially improving communication between all involved.	
9	Ribeirão Preto/SP	2007	Home visit [3, 16]	An instrument for conducting home care, consisting of systematic actions to enable care to people with some level of change in health (physical or emotional dependence) or to perform activities related to health programs.	Organization of Assistance
10	Rio de Janeiro/RJ	2007	Administrative Order System (held in CADJ) [17-19]	Receive subpoenas concerning the public defender and the public prosecutor in health actions, comply with court decisions and organize and control the stock of medicines and supplies intended to comply with court orders, to avoid the use of emergency bid waiver for the acquisition of these items and expedite the rendering of services in court cases.	Administrative proceeding
11	São Paulo/SP	2007	Technical Report CODES [10, 11, 20]	Technical report that explains in easy-to-understand language the disease that affects the litigant, the relevance of the drug therapy being claimed, and the therapeutic options or alternatives provided by SUS.	Defense of the public authority
12	São Paulo/SP	2007	Pharmaceutical Screening [11, 21]	Reduce lawsuits pending through an out-of-court dispute settlement mechanism.	Alternative dispute resolution
13	Vitória/ES	2007	Reference Centers (Clinical Audit) [12, 13]	Establish a system for regulating the prescription and promotion of rational use of medicines provided by State Pharmacies of Exceptional Medicines.	Organization of Assistance
14	Vitória/ES	2007	Permanent Intersectoral Forum of Pharmaceutical Assistance of Espírito Santo (FIPAFES) [12, 13, 22]	Conduct the implementation of the Pharmaceutical Policy of the State of Espírito Santo, aiming at ensuring its highest purpose of providing equitable access to quality essential and exceptional medicines, at all levels of health care, taking care to promote their rational use and humanization of the care provided to its citizens.	Organization of Assistance
15	Vitória/ES	2007	Nucleus of Court Orders (2017) [12]	Meet legal demands, as well as guide and provide subsidies to the Public Prosecution Service, the Prosecutor's Office, the Attorney's Office and the Judiciary Power on drug issues.	Compliance with court orders
16	Vitória/ES	2007	Citizen Pharmacy Project [6, 12]	Expand access to medicines and improve the quality of services offered to users of high-cost state drugs, because the conditions of old pharmacies were deficient.	Organization of Assistance
17	Rio de Janeiro/RJ	2007	Judicial Demand Response Center (CADJ) (Single or Warrant Center) [19, 23]	Attempt to solve the demands that sought the supply of medicines within the competence of the Municipality or the specialized component and competence of the State.	Compliance with court orders
18	São Paulo/SP	2008	Intelligence Core [24]	Map all actions and study the authors involved in this intricate phenomenon called judicialization through software. The purpose of the joint action is to add value to the information collected so far and to identify the reason for this growing demand that plagued the state.	Organization of Assistance

No.	County/ state	Year of implementation	Strategy name	Objetives	Strategy Category
19	São Paulo/SP	2008	8th Department Attorney General's Office, Attorney General's Office of the State of São Paulo (PJ 8) [10, 11, 25]	Improve the performance of the State Attorney General's Office with the Superior Court of Justice and the Federal Supreme Court.	Defense of the public authority
20	Belo Horizonte/ MG	2009	Judicial Judgments Action via State Health Fund (FES MG) [15, 26]	Own rubric for meeting judicial "sentences".	Compliance with court orders
21	Brasília/DF	2009	National Pharmaceutical Assistance System (Hórus System) [27, 28]	Qualify the management of Pharmaceutical Assistance in the three spheres of the SUS, contributing to the expansion of access to medicines and the qualification of health care provided to the population.	Computerized Information System
22	Mauá/SP	2009	Pharmaceutical Assistance Lawsuit Sector [29]	Planning and reorganization of actions.	Compliance with court orders
23	Natal/RN	2009	Interinstitutional Committee on Administrative Resolutions on Health Demands (CIRADS) [30-32]	Serve the citizen through the implementation of public health policies already existing in the SUS, promoting administrative conciliation to avoid unnecessary judicialization of health actions or promote conciliation in actions already filed and where there is the possibility of meeting the claim by the Government , as well as seek general solutions to health issues in order to benefit those assisted by SUS.	Alternative dispute resolution
24	Porto Alegre/RS	2009	Health-Focused Systemic Planning Action [33, 34]	Facilitate access to the health system and ensure effectiveness to this right through extrajudicial solutions.	Organization of Assistance
25	Rio de Janeiro/RJ	2009	Technical Advisory Center (NAT) [17, 19, 35, 36]	Assist magistrates in making decisions on the subject; provide technical subsidies to magistrates and public defenders in lawsuits aimed at compelling the State of Rio de Janeiro to provide medical supplies, medicines or treatments.	Technical support to the judiciary
26	São Paulo/SP	2009	Administrative Assistance [11, 25, 37, 38]	Standardize the receipt by SES of administrative requests for drugs not standardized by SUS.	Administrative proceeding
27	Três Rios/RJ	2009	Technical support center in health for the Attorney Office (Napass) (Technical Chamber) [39, 40]	Management tool seeking to contain the amount and cost of judicial demands but ensuring the exercise of the right to health by citizens.	Defense of the public authority
28	Porto Alegre/RS	2010	State Executive Committee of Health [33, 34]	Significantly reduce the number of actions that claim health supplies or services, because patient care in their right to health should be given less severely to public entities, so lacking resources and constantly expanding institutional demands.	State Health Committee

No.	County/ state	Year of implementation	Strategy name	Objetives	Strategy Category
29	São Paulo/SP	2010	CODES System (S-CODES) (20, 41)	Control and management of lawsuits.	Computerized Information System
30	Araguaína/TO	2011	Araguaína Municipal Technical Support Center (Municipal NAT) (42-45)	Acting as a means of administrative solution by stimulating dialogue between the Executive and Judiciary, the Public Prosecution Service, the Public Defender's Office and Civil Society (users), preventing and avoiding the judicialization of the demands on health care, is also a support tool for the Judiciary to issue decisions to construct technical opinions on health law and public policies that involve the right to public health.	Administrative proceeding / Alternative Dispute Resolution
31	Araguaína/TO	2011	Administrative Conflict Resolution (43, 44)	Giving voice to SUS users, welcoming their manifestations, providing individualized answers that can meet the punctuated needs, and thus seeks to contribute information that can support management in the process of continuous improvement of health care in Araguaína.<br>Avoid judicialization and subsidize the judges in decisions about lawsuits involving health care.<br>Encourage, educate and make citizens aware, adding pedagogical effects, in order to directly access the health system managed by the local Executive, as services are available. On the other hand, demand from the health manager, the effective implementation and execution of the public policies provided for health.	Administrative proceeding
32	Belo Horizonte/ MG	2011	Technical Cooperation Specialized Health Defender's Office (15, 46, 47)	Consistently act to meet the demands of granting medicines, procedures and medical and health supplies, in face of SUS, prioritizing the administrative solution through a multidisciplinary team, reducing the number of judicial demands, and promoting the integration between entities and to avoid lawsuits involving health benefits.	Alternative dispute resolution
33	Brasília/DF	2011	National Commission for the Incorporation of Technologies for SUS (Conitec) (48)	Provide clarifications to prosecutors, still in the civil inquiry phase, about the availability of health technologies by the SUS, which can sometimes prevent the filing of new lawsuits.<br>Provide technical grants to the Union Attorney General for the defense of the Union in lawsuits.<br>Provide Judges with technical support so that they may have information to enable them to make decisions on requests for injunctions in lawsuits.	Pharmacy and Therapeutic Committee
34	Brasília/DF	2011	Judicialization Center of the Federal District Department of Health (49)	Improve the provision of services to individuals who require SUS care and who have not yet resorted to the judiciary.	Administrative proceeding

No.	County/ state	Year of implementation	Strategy name	Objetives	Strategy Category
35	Campo Grande/MS	2011	Technical Support Center (NAT-Jus 2016) [50]	Advise the State Judiciary, with technical information, in the demands related to the supply of medicines, examinations, hospitalizations and other treatments in the face of SUS.	Technical support to the judiciary
36	Curitiba/PR	2011	Paraná Executive Health Committee [7]	Subsidize magistrates and other legal operators to ensure greater efficiency in solving judicial demands involving health care.	State Health Committee
37	Recife/PE	2011	Health Technical Assistance Center (NAT-JUS) of Pernambuco [51]	Promote the elucidation of the demands arising from lawsuits involving public health issues, acting as a support tool for magistrates, to allow further clarification on the subject.	Technical support to the judiciary
38	São Luís/MA	2011	Executive Committee of the State of Maranhão [52, 53]	Monitor health demands in the state of Maranhão and by proposing concrete and normative measures to subsidize magistrates.	State Health Committee
39	Belo Horizonte/ MG	2012	Health Mediation [54]	Discuss the various collective health problems, at the micro or macroregional level, in order to allow a democratic interaction between the participants. Support prosecutors working in health advocacy and other actors in solving complex health demands, with emphasis on the adoption of preventive measures of collective reach, seeking to build solutions through consensus and replacing, where possible, judicialization of policy of health.	Alternative dispute resolution
40	Belo Horizonte/ MG	2012	Technology Assessment Center of Clinics Hospital (2012 to 2014) [55]	Provide critical analysis of new technologies and processes, their clinical value, and their possibilities for incorporation into clinical practice, and to promote evidence-based health practice through study, practice, and knowledge dissemination, as well as the culture of improvement cycles through shared learning and leadership.	Technical support to the judiciary
41	João Pessoa/PB	2012	Technical Health Chamber (CTS) [50]	Subsidize the State Judiciary with technical information in the demands related to the supply of medicines, orthosis and prosthesis, examinations, hospitalizations and clinical or surgical treatments of SUS.	Technical support to the judiciary
42	Lages/SC	2012	Health Demand Conciliation Center [43, 56-58]	Pre-procedural conciliation center is a conflict resolution model that aims to provide an alternative to the judicialization of health demands, to effectively and effectively meet drug requests, as well as examinations, procedures and / or surgical treatments, supplies and others by SUS users not yet judicialized.	Alternative dispute resolution
43	Natal/RN	2012	SUS Mediated Natal [59-63	Guarantee greater effectiveness to public health policies in the state of Rio Grande do Norte, avoid judicial demands and ensure access to the SUS unsufficient users to medicines and medical procedures under the responsibility of the State of Rio Grande do Norte, provided before the SUS.	Alternative dispute resolution

No.	County/ state	Year of implementation	Strategy name	Objetives	Strategy Category
44	Palmas/TO	2012	Executive Committee for the Monitoring of Health Actions in the State of Tocantins (CEMAS-TO) [42, 45, 64]	Coordinate and execute actions of specific nature, considered relevant within the scope of care and health care services, and provide the broad and open dialogue in favor of prior composition for the solution of individualized and collective demands, with the purpose of avoiding judicialization. Monitor lawsuits involving the provision of health care, such as the provision of medicines and products, examinations, surgical interventions, provision of ICU beds, etc.	State Health Committee
45	Porto Alegre/RS	2012	Utilization of the Medication Administration System (AME) [34, 65]	Record all drug deliveries granted by court orders.	Computerized Information System
46	Porto Alegre/RS	2012	Judicial Medical Department (DMJ) [66]	Conduct examinations and prepare technical opinions to instruct judicial and Administrative proceedings.	Technical support to the judiciary
47	Rio de Janeiro/RJ	2012	Health Dispute Resolution Chamber (CRLS) [17, 19, 59, 67	Promote the care of parties assisted by the DPE and the DPU and demanding the provision of health services, in order to avoid the filing of lawsuits, seeking administrative solution for offering medication, scheduling surgical or clinical procedure, hospitalization or medical examination.	Alternative dispute resolution
48	Salvador/BA	2012	Judiciary Technical Support Center (NAT-JUS) (2017) [68-70]	Exclusive assignment of providing expert evidence-based health information to judges to assist them in decision-making involving clinical and contractual or public policy relevance, as appropriate, of medicines, products, therapeutic inputs, surgical procedures and diagnostic exams, hospitalizations or the like, related to the public sector (SUS) or supplementary health (insurance or health plans).	Technical support to the judiciary
49	São Paulo/SP	2012	Pharmacology Committee of the São Paulo State Secretariat of Health [37, 38, 71]	Advise the Holder in the formulation of guidelines for the selection, standardization, prescription, acquisition, distribution, dispensation and pharmacotherapeutic follow-up within the SUS of the State of São Paulo, in accordance with the National Medicines Policy.	Pharmacy and Therapeutic Committee
50	São Paulo/SP	2012	Pharmaceutical Screening at the Small Claims Courts (JEFAZ) [25, 72]	To make a prior analysis of the process, by SES-SP technicians at the courts of the special Treasury of the Public, in order to identify other means for the patient, other than judicialization, reduce unnecessary lawsuits, increase rational use of medicines with greater safety and effectiveness and reinsert the patient in the SUS as much as possible.	Alternative dispute resolution
51	Brasília/DF	2013	District Permanent Chamber of Health Mediation (Camedis) [43, 59, 62, 73]	Seek solutions to the demands for health services and products, in order to avoid lawsuits or to propose solutions to those in progress and strengthen the extrajudicial mechanisms for the realization of the right to health.	Alternative dispute resolution

No.	County/ state	Year of implementation	Strategy name	Objetives	Strategy Category
52	Franca/SP	2013	Transfer of Citizens from Attendance Pharmacy to Pharmaceutical Assistance Specialty Pharmacies [74]	Demonstrate the benefits of patient care through Pharmaceutical Assistance Specialized Drugstores over the legal pathway. Promote rational use of medicines by providing them with guidance, safety and therapeutic efficacy and reducing expenses with judicialization.	Organization of Assistance
53	Maceió/AL	2013	Interinstitutional Center for Health Judicialization (NIJUS) [75, 76]	Assist the Secretaries of the State of Health and Municipal Health of Maceió, respectively, in the pertinent analyzes of the lawsuits that relate strictly to the provision of health services within the SUS, in Alagoas.<br>Promote studies, analysis, seminars and other events related to the processing and / or processing of lawsuits in the Health area in Alagoas.<br>Assist the DPU and / or the State of Alagoas, upon request, as well as the Federal and State Judiciary, respectively, regarding the processes related to health issues, within the SUS, in Alagoas.<br>Cooperate with the technical areas of the mentioned secretariats, strictly regarding lawsuits related to health, under the SUS, in Alagoas.	Compliance with court orders / Administrative proceeding
54	Palmas/TO	2013	State NAT (State NAT-JUS -TO) [42, 45, 77]	Provide technical subsidies to the Tocantinense Magistrates in public health demands aimed at actions and services offered by the SUS, in order to improve the technical knowledge of the Magistrates, and thus give more security in decision making through the exchange of information. institutional information.	Technical support to the judiciary
55	Rio de Janeiro/RJ	2013	Health Chamber System [19]	It serves as a management aid tool.	Computerized Information System
56	Belém/PA	2014	Interinstitutional Committee on Administrative Resolution of Health Demands (CIRADS) [78]	Analyze the specific cases in which the individual, assisted or not by the SUS, claims not to have received proper care, in order to identify the causes of the problem and forward solutions, for science and confirmation of the terms presented. To act in the administrative care of the claim, avoiding its judicialization, as well as in the health treatments (supply of medicines, supplies, materials and health services) provided for in the SUS's competences and has not been provided, as well as in those hypotheses in which, for some reason, the doctor indicated different treatment from those offered by SUS.	Alternative dispute resolution
57	Belo Horizonte/ MG	2014	Judicialization of Health Care Assistance Center (NAJS) [26, 54, 79]	Propose and implement methods and work routines that speed up the execution of the demands of your area of expertise.<br>Promote actions to ensure compliance with a court order determining the supply of medicines, supplies and medical and / or hospital procedures.<br>Perform, within its sphere of attributions, other duties assigned to it.	Organization of Assistance
58	Recife/PE	2014	Nucleus of Lawsuits (NAJ) [51]	Speed up the fulfillment of court orders and responses to administrative requirements in the Health area in order to respond quickly to patients	Compliance with court orders

No.	County/ state	Year of implementation	Strategy name	Objetives	Strategy Category
				who seek, through the courts, access to medicines, supplies and equipment, hospitalizations, prostheses and orthotics, as well as other network public treatments.	
59	Uberlândia/ MG	2014	Interinstitutional Committee on Health Demand Resolution (CIRDS) Uberlândia (80)	To express previously, that is, before the judicial phase, about the requests regarding the supply of medicines, aiming at the out-of-court settlement under the SUS guidelines.	Alternative dispute resolution
60	Cantagalo/RJ	2015	Extrajudicial Service in Public Health (81)	Serve the population that seeks access to public health services in the city of Cantagalo / RJ.	Alternative dispute resolution
61	Curitiba/PR	2015	Health Attorney Paranaense (7, 82)	To act in Defense of the public authority in all demands involving the right to health in the suits filed in Curitiba, whether they were filed in State or Federal Justice.	Defense of the public authority
62	Macapá/AP	2015	Ombudsman's Office and Technical Support of the Amapá State Secretariat of Health (NOAT) (83)	Addressing health demands and avoiding lawsuits, as well as providing channels for easy and agile contact, even those living in places with poor access to information.	Administrative proceeding
63	Maceió/AL	2015	State Attorney General's Office (PGE): Bids, Contracts, and Agreements Subunit (84)	Reduce response time to comply with lawsuits.	Defense of the public authority
64	São Luís/MA	2015	Health Care Portal (PAS) (85, 86)	Avoid judicialization and seek administrative solutions regarding access to medicines, examinations, hospitalizations, treatments and hospital transfers, using mediation, conciliation and information.	Alternative dispute resolution
65	Vitória/ES	2015	Judicialization Strategic Management Project (87)	Structuring the service of judicial demands of SESA, to systematize the practice and generate strategic actions to cope with justifiable and unjustifiable demands, with the strengthening of dialogue with the legal operators and the external actors of society that demand the Secretariat of State in different ways. Health on Judicialization issues.	Organization of Assistance
66	Fortaleza/CE	2016	Judiciary Technical Support Center (NAT-JUS) (88, 89)	Respond to requests for clarification in lawsuits involving the provision of health care in the SUS.	Technical support to the judiciary
67	Macapá/AP	2016	Nucleus of Judicial Technical Support for Health Demands (NAT-JUS) (90, 91)	Provide multidisciplinary expertise to judges in decisions involving public or private health cases and reduce judicialization.	Technical support to the judiciary
68	Maceió/AL	2016	Technical Health Chamber (CTS) (92)	Provide specialized, non-binding information to assist judges in their decisions involving the technical or contractual relevance of benefits,	Technical support to the judiciary

No.	County/ state	Year of implementation	Strategy name	Objetives	Strategy Category
				medications, surgical procedures, diagnoses, hospitalizations or the like, related to the SUS sector or supplementary health.	
69	Natal/RN	2016	NAT-Jus Rio Grande do Norte [93]	Responsible for the elaboration of technical and specialized documents in the health area, to assist magistrates in the formation of a value judgment, in the consideration of clinical issues presented in the lawsuits, involving the public health assistance, according to Recommendation no. 31/2010 and Resolution 238/2016, both of the National Council of Justice.	Technical support to the judiciary
70	Palmas/TO	2016	Palmas Support Center (NAT-Jus) [45]	Reduce the irrational spending of public resources resulting from judicialization, committed to improving the quality of public health service delivery in the city of Palmas, with a view to SUS sustainability, and ensuring greater speed and bureaucracy to the public service, providing greater efficiency of the public service to the citizen.	Technical support to the judiciary
71	São Paulo/SP	2016	Pharmaceutical and Nutrition Screening and Guidance Services (Acessa SUS) [94-96]	Reduce unnecessary lawsuits and provide information on alternative medication and treatments already existing in the public network, through direct service to the population and demands from the state prosecutor, public defender and judiciary.	Administrative proceeding / Alternative dispute resolution
72	Vitória/ES	2016	Extrajudicial Health Procedure (PES System) [97]	Administrative resolution without the need to proceed with judicialization.	Computerized Information System
73	Belém/PA	2017	Judiciary Technical Support Center (NAT-JUS Pará) [78]	Organize and promote the fulfillment of judicial demands related to public health in the State of Pará and provide technical support to magistrates of first and second instance in demands involving the right to public health.	Technical support to the judiciary
74	Brasília/DF	2017	National Register of Notes and Technical Information (e-NAT-Jus) [98]	Subsidize judges from across the country in health lawsuits and avoid conflicting opinions between different NATs through a tool with technical and scientific evidence.	Computerized Information System
75	Campo Grande/MS	2017	State Committee of the Judiciary Forum for Health [5, 99]	To advise and suggest to Federative Entities, Magistrates, the medical profession and other legal operators involved in public health, measures to alleviate the problem of judicialization of health, as well as to strengthen ties with the main political spheres, through judicial policy aimed at management. from the health area.	State Health Committee
76	Palmas/TO	2017	Nucleus of Lawsuits [42]	Exclusive sector for the Administrative proceeding to comply with the lawsuits received by SES.	Compliance with court orders
77	Campo Grande/MS	2018	Compliance with small health court rulings as specified [99, 100]	Comply with the injunction or judgment by judicial deposit of the amount necessary for the patient to purchase the product directly, for a period of up to 180 (one hundred and eighty) days.	Compliance with court orders
78	Vitória/ES	2018	Electronic Portal of Judicial Subpoena [101, 102]	Give more speed to compliance with lawsuits, centralizing and standardizing the receipt of subpoenas at SESA.	Computerized Information System

Legends: AL - Alagoas; AP - Amapá; BA - Bahia; CE - Ceará; CODES - Coordination of SUS Strategic Demands; DF – Federal District; DPE - State Public Defender's Office; DPU - Federal Public Defender's Office; ES - Espírito Santo; MA - Maranhão; MG - Minas Gerais; MS - Mato Grosso do Sul; NAT - Technical Support Center; PA - Pará; PB - Paraíba; PE - Pernambuco; PR - Paraná; REMEME - State List of Medicines; RJ - Rio de Janeiro; RN - Rio Grande do Norte; RS - Rio Grande do Sul; SC - Santa Catarina; SES ou SESA - State Health Secretariat; SP - São Paulo; SUS - Unified Health System; TO- Tocantins; ICU - Intensive care unit.

Data checked in March 2019.

References

1. Perin IG, Tassinari MTR, Santos MG, Santos IES, Larogian APA, Teixeira VM, et al. Desafios e avanços da comissão de análise de solicitações especiais visando a equidade e o aprimoramento da gestão do SUS. In: Unidade de Desenvolvimento e Melhoria das Organizações, editor. Prêmio Mario Covas, 5ª edição, ciclo 2008; Theatro Municipal de São Paulo. São Paulo: Secretaria Estadual de Planejamento e Gestão; 2008.

2. Boldrin PHM. A efetividade da Comissão de Análise de Solicitações Especiais da judicialização das políticas de saúde referentes às demandas ajuizadas pela Defensoria Pública do Estado de São Paulo - Regional Ribeirão Preto. Ribeirão Preto: Universidade de São Paulo; 2014.

3. Secretaria da Saúde do Município de Ribeirão Preto. Serviço de Atenção Domiciliar – SAD. Ribeirão Preto: Prefeitura da cidade de Ribeirão Preto; 2017 [cited 2019 abril 6]. Available from: https://www.ribeiraopreto.sp.gov.br/ssaude/programas/sad/i16indice.php.

4. Mauad MLME, Tanaka NYY, Fazan WC. Protocolo inicial da comissão de análise de solicitações especiais para dieta enteral. In: Coordenação-Geral da Política de Alimentação e Nutrição, editor. II Mostra de Alimentação e Nutrição do SUS I Seminário Internacional de Nutrição na Atenção Primária: trabalhos selecionados para apresentação oral. Comunicação e Educação em Saúd. Brasília: Ministério da Saúde; 2009. p. 122.

5. Nantes LFL, Dobashi BF. A experiência da secretaria estadual de saúde de Mato Grosso do Sul no enfrentamento das ações judiciais em saúde. In: CONASS, editor. Direito à saúde. Para Entender a Gestão do SUS - 2015. 1ª ed. Brasileiro: CONASS; 2015. p. 113.

6. Conselho Federal de Farmácia. Experiências exitosas de farmacêuticos no SUS. 2013;ano I(1):82.

7. Pontarolli DRS, Silva GRPP, Strapasson GC. O enfrentamento das demandas judiciais por medicamentos na secretaria de Estado da saúde do Paraná. In: CONASS, editor. Direito à saúde. Para Entender a Gestão do SUS - 2015. 1ª ed. Brasília: CONASS; 2015. p. 113.

8. Bahia GOM, Lamb L, Nicoletti RHA, Cataneli RCB, Luiz VR. A política de medicamentos no Estado do Paraná. In: Saúde. CONASS, editor. Relatório final do I encontro do CONASS para troca de experiências. CONASS Documenta. Brasília: CONASS; 2005. p. 140.

9. Secretaria de Estado da Saúde do Paraná, Superintendência de Gestão de Sistemas de Saúde. Assistência Farmacêutica. Curitiba: Secretaria de Estado da Saúde do Paraná; 2008.

10. Sanchez LA, Santos LN, Gomes RAC, Chieffi AL, Souza CZB, Camargo CR, et al. Uma parceria inovadora para fazer frente ao fenômeno da "judicialização das políticas públicas de saúde": a otimização da gestão pública. In: Unidade de Desenvolvimento e Melhoria das Organizações, editor. Prêmio Mario Covas, 5ª edição, ciclo 2008; Theatro Municipal de São Paulo. São Paulo: Secretaria Estadual de Planejamento e Gestão; 2008.

11. Yoshinaga JY. Judicialização do direito à saúde: a experiência do estado de São Paulo na adoção de estratégias judiciais e extrajudiciais para lidar com esta realidade. Salvador: Revista Eletrônica sobre a Reforma do Estado; 2011 [cited 2019 abril 6]. [Available from: http://www.direitodoestado.com.br/rere/edicao/24.

12. Tavares GRP, Silva DM, Barcelos PC, Ribeiro C, Moreira GL. Diagnóstico das ações judiciais direcionadas à secretaria de Estado da saúde do Espírito Santo. In: Conselho Nacional de Secretários de Estado da Administração, editor. III Congresso Consad de Gestão Pública; 15 a 17 de março de 2010. Centro de Convenções Ulisses Guimarães, Brasília/DF. Brasília: Consad; 2010. p. 31.

13. Tavares GRP, Silva DM, Bernardos A. Sistema de regulação da dispensação de medicamentos excepcionais. In: Conselho Nacional de Secretários de Estado da Administração, editor. II Congresso Consad de Gestão Pública; 6 a 8 de maio de 2009. Centro de Convenções Ulysses Guimarães, Brasília-DF. Brasília: CONSAD; 2009. p. 24.

14. Pereira LR, Carneiro R. Judicialização da saúde e mudanças organizacionais: o impacto das decisões judiciais na estrutura da secretaria de Estado de saúde de Minas Gerais (SES-MG). V Encontro da Divisão de Administração Pública/APB da ANPAD - EnAPG; 18 a 20 de novembro de 2012, Salvador/BA Maringá: Associação Nacional de Pós-Graduação e Pesquisa em Administração; 2012.

15. Pereira LR. Judicialização da saúde e mudanças organizacionais: o impacto das decisões judiciais na estrutura da Secretaria Estadual de Saúde de Minas Gerais (SES-MG). Belo Horizonte: Escola de Governo Professor Paulo Neves de Carvalho; 2012.

16. Chayamiti EMPC, Coordenadora. Manual do serviço de atenção domiciliar. 2ª revisão ed. Ribeirão Preto: Secretaria municipal da saúde de Ribeirão Preto; 2018. 64 p.

17. Souza AM. A atuação em rede de instituições governamentais na resolução de conflitos sobre demandas sanitárias no Rio de Janeiro. Rio de Janeiro: Fundação Getúlio Vargas; 2016.

18. Teixeira MF. Criando alternativas ao processo de judicialização da saúde: o sistema de pedido administrativo, uma iniciativa pioneira do estado e município do Rio de Janeiro". Rio de Janeiro: Fundação Oswaldo Cruz; 2011.

19. Guimarães RCM, Palheiro PHD. Medidas adotadas para enfrentar a judicialização na secretaria de saúde do Estado do Rio De Janeiro e a experiência da Câmara de Resolução de Litígios de Saúde. In: CONASS, editor. Direito à saúde. Para Entender a Gestão do SUS – 2015. 1ª ed. Brasília: CONASS; 2015. p. 113.

20. Naffah-Filho M, Chieffi AL, Correa MCMMA. S-Codes: um novo sistema de informações sobre ações judiciais da Secretaria de Estado da Saúde de São Paulo. BEPA. 2010;7(84):12.

21. Casal VAS, Motta LJ, Carvalho A, Maximiano VAZ. Obtenção de medicamentos extrajudicialmente. VI Prêmio Innovare: Justiça Rápida e Eficaz; 17 de dezembro de 2009. Supremo Tribunal Federal, Brasília. Rio de Janeiro: Instituto Innovare; 2009.

22. Governo do Estado do Espírito Santo. Decreto n° 1956-R, de 07 de novembro de 2007: Aprova a Política Farmacêutica do Estado do Espírito Santo e dá outras providências. Vitória: Gabinete do governador; 2007. p. 5.

23. Junior PBR. A judicialização do acesso a medicamentos: A perspectiva da secretaria municipal de saúde do Rio de Janeiro. Rio de Janeiro: Fundação Getulio Vargas - Escola Brasileira de Administração Pública e de Empresas; 2008.

24. Ungaro G. O combate às fraudes judiciais em ações para fornecimento de medicamentos no Estado de São Paulo. VIII edição do Prêmio Innovare; 15 de dezembro de 2011. Tribunal Federal de Justiça, Brasília. Rio de Janeiro2011.

25. Toma TS, Soares AC, Siqueira PSFd, Domingues R. Estratégias para lidar com as ações judiciais de medicamentos no estado de São Paulo. Cad Ibero-Amer Dir Sanit. 2017;6(1):19.

26. Simões FHS. Judicialização do acesso ao tratamento do diabetes no Estado de Minas Gerais. Belo Horizonte: Universidade Federal de Minas Gerais; 2015.

27. Costa KS, Nascimento-Jr JM. HÓRUS: inovação tecnológica na assistência farmacêutica no sistema único de saúde. Revista de Saúde Pública. 2012;46:91-9.

28. Ministério da Saúde, Assistência Farmacêutica. Sistema Hórus. Brasília: Ministério da Saúde; 2013 [cited 2019 abril 2]. Available from: http://portalms.saude.gov.br/assistencia-farmaceutica/sistema-horus/sobre-o-sistema.

29. Garofalo G, Baseio C, Campos L, Alonso MS. Atenção farmacêutica na diminuição da prescrição e do uso irracional de medicamentos na judicialização do SUS. Prêmio Nacional de Incentivo à Promoção do Uso Racional de Medicamentos – 2011/Ministério da Saúde, Secretaria de Ciência, Tecnologia e Insumos Estratégicos, Departamento de Ciência e Tecnologia. Brasília: Ministério da Saúde; 2012. p. 102.

30. Queiroz CC. A mediação como instrumento concretizador do direito fundamental à saúde: uma alternativa à judicialização de conflitos. Natal: Universidade Federal do Rio Grande do Norte; 2013.

31. Pinheiro TP. Comitê Interinstitucional de Resolução Administrativa de Demandas da Saúde (CIRADS). VII edição do Prêmio Innovare: Justiça sem burocracia e acesso do preso à Justiça; 3 de dezembro de 2010. Tribunal Federal de Justiça, Brasília. Rio de Janeiro: Instituto Innovare; 2010.

32. Advocacia Geral da União. CIRADS. Brasília: Advocacia-Geral da União; 2009 [updated 2013; cited 2019 abril 6]. Available from: http://www.agu.gov.br/page/content/detail/id_conteudo/97046.

33. Schulze M. A desjudicialização da saúde no Rio Grande do Sul: cabal redução do número das ações ativas e estratégia de interiorização. In: Santos AO, Lopes LT, editors. Boas Práticas e Diálogos Institucionais. Coletânea Direito à Saúde. 3. 1ª ed. Brasília: CONASS; 2018. p. 292.

34. Comitê Executivo do Fórum Nacional do Judiciário para Saúde do Conselho Nacional de Justiça. Ação de planejamento e de gestão sistêmicos com foco na saúde. Porto Alegre: Comitê Executivo do Rio Grande do Sul; 2015. 287 p.

35. Castro KRTR. Os juízes diante da judicialização da saúde: o NAT como instrumento de aperfeiçoamento das decisões judiciais na área da saúde. Rio de Janeiro: Fundação Getúlio Vargas; 2012.

36. Silva MV. O processo decisório judicial e a assessoria técnica: a argumentação jurídica e médico-sanitária na garantia do direito à assistência terapêutica no Sistema Único de Saúde. Rio de Janeiro: Fundação Oswaldo Cruz; 2012.

37. Secretaria de Estado da Saúde do Estado de São Paulo. Comissão de Farmacologia. São Paulo: Secretaria de Estado da Saúde; 2019 [cited 2019 abril 6]. Available from: http://saude.sp.gov.br/ses/perfil/gestor/comissao-de-farmacologia/.

38. Toma TS, Soares AC, Bortoli MC, Pirotta KCM, Venâncio SI, Derbli M, et al. Avaliação de tecnologias e inovação em saúde no SUS: desafios e propostas para a gestão. São Paulo: Instituto de Saúde; 2015. 344 p.

39. Bandeira LM, Bandeira RC, Costa CA, Santos JBFMS, Silva HA, Furtado EAV. A atuação do Núcleo de Apoio à Procuradoria Adjunta de Suporte à Saúde no município de Três Rios como um norte para a contenção saudável da judicialização em saúde. Rio de Janeiro: IdeiaSUS - Banco de Práticas e Soluções em Saúde e Ambiente; 2019 [cited 20191 abril 6]. Available from: http://www.ideiasus.fiocruz.br/portal/index.php/banco-de-praticas.

40. Costa CA. Gestão de pessoal e educação permanente em saúde pública como viés para redução do ativismo judicial. Rio de Janeiro: IdeiaSUS - Banco de Práticas e Soluções em Saúde e Ambiente; 2019 [cited 2019 abril 6]. Available from: http://www.ideiasus.fiocruz.br/portal/index.php/banco-de-praticas/busca-avancada.

41. Siqueira PSF, Martins MA, Domingues R. S-CODES: Sistema informatizado para o trato da judicialização. In: Santos AO, Lopes LT, editors. Boas Práticas e Diálogos Institucionais. Coletânea Direito à Saúde. 3. 1ª ed. Brasília: CONASS; 2018. p. 292.

42. Farias DR. Judicialização da saúde: aspectos processuais e institucionais na efetivação do direito à saúde pública no Estado do Tocantins. Palmas: Universidade Federal do Tocantins; 2016.

43. Asensi FD, Pinheiro R, Coordenadores. Judicialização da saúde no Brasil: dados e experiência. Brasília: Conselho Nacional de Justiça; 2015. 142 p.

44. Henrique MC, Brito JOB, Mel MDSM. Eficiência na solução das demandas de judicialização da saúde na Comarca de Araguaina-TO. Cad IberAmer Direito Sanit. 2013;2(2):19.

45. CEMAS. Comitê Executivo para Monitoramento das Ações da Saúde no Estado do Tocantins – CEMAS. Palmas: Tribunal de Justiça do Estado do Tocantins; 2019 [cited 2019 abril 6]. Available from: http://wwa.tjto.jus.br/saude/.

46. Reis BB. Defensoria especializada da saúde. In: Associação Nacional de Defensores Públicos, editor. Concurso de práticas exitosas 2011 X Congresso Nacional de Defensores Públicos (ANADEP); 15 a 18 de novembro, Centro de Convenções de Natal -RN. Brasília: ANADEP; 2011. p. 16.

47. Secretaria Estadual de Saúde. SES-MG e Defensoria Pública estabelecem parceria pioneira para agilizar assistência farmacêutica no Norte de Minas. Belo Horizonte: Conselho Regional de Farmácia do Estado de Minas Gerais; 2016 [updated 08/03/2016; cited 2019 abril 6]. Available from: http://www.crfmg.org.br/site/Noticias/SES-MG-e-Defensoria-Publica-estabelecem-parceria-pioneira-para-agilizar-assistencia-farmaceutica-no-Norte-de-Minas.

48. Simabuku EMG, Catanheide ID, Biella CA, Rabelo RB, Santos VCC, Petramale CA. Comissão nacional de incorporação de tecnologias no SUS e a judicialização do acesso à saúde. Revista Eletrônica Gestão & Saúde. 2015;6(Supl. 4):18.

49. Conti MA. Avaliação das demandas judiciais por acesso a medicamentos no Distrito Federal. Brasília: Universidade de Brasília. Faculdade de Ciências da Saúde; 2013.

50. Costa IC. Judicialização da saúde e a Câmara Técnica de Saúde no tribunal de justiça da Paraíba. Guabira: Centro de Humanidades da Universidade Estadual da Paraíba; 2014.

51. Barros LD. Judicialização do direito à saúde: uma análise acerca do fornecimento judicial de medicamentos no Estado de Pernambuco. Recife: Universidade Federal de Pernambuco; 2016.

52. Diniz IM. Direito à saúde e judicialização: uma análise da atuação do Conselho Nacional de Justiça no aprimoramento da prestação jurisdicional nas demandas de saúde. São Luís: Universidade Federal do Maranhão; 2015.

53. Tribunal de Justiça do Maranhão. Portaria-GP nº 6832018. Dispõe sobre a composição da Comissão do Comitê Estadual de Saúde do Estado do Maranhão. São Luís: Tribunal de Justiça do Maranhão; 2018. p. 1.

54. Assis G. Mediação sanitária: direito, saúde e cidadania. In: CONASS, editor. Direito à saúde. Para Entender a Gestão do SUS - 2015. 1ª ed. Brasília: CONASS; 2015. p. 113.

55. Duarte VG. Arranjos e diálogos institucionais para enfrentamento da judicialização da saúde: uma análise dos modelos de assessoramento técnico (NAT’s). Limeira: Universidade Estadual de Campinas; 2017.

56. Orsatto SD. Núcleo de conciliação de demandas em saúde. XI edição do Prêmio Innovare; 16 de dezembro de 2014. Supremo Tribunal Federal, Brasília. Rio de Janeiro: Instituto Innovare; 2014.

57. Prefeitura Municipal de Lages. Núcleo de Medicamentos. Município e Tribunal de Justiça firmarão convênio. Lages: Prefeitura de Lages; 2015 [updated 23/03/2015; cited 2019 abril 3]. Available from: http://www.lages.sc.gov.br/noticia/5800/municipio-e-tribunal-de-justica-firmarao-convenio/.

58. Vasconcellos J. Núcleo de conciliação de Lages/SC soluciona 90% dos conflitos na saúde. Brasília: CNJ; 2015 [updated 08/05/2015; cited 2019 abril 3]. Available from: http://cnj.jus.br/noticias/cnj/79305-nucleo-de-conciliacao-de-lages-sc-soluciona-90-dos-conflitos-na-saude.

59. Sant’Ana RN. A judicialização como instrumento de acesso à saúde: propostas de enfretamento da injustiça na saúde pública. Brasília: Centro Universitário de Brasília; 2017.

60. Júnior LMRR. A mediação como instrumento de efetivação do direito à saúde: análise dos relatórios de antendimentos realizados pelo programa "SUS Mediado", no ano de 2014. Recife: Universidade Católica de Pernambuco; 2017.

61. Ringeisen ATS. Mediação de conflitos no sistema único de saúde: visões e práticas de uma experiência no município de Natal/RN. Natal: Universidade Federal do Rio Grande do Norte; 2016.

62. Cotrim TP. Mediação como instrumento de ação pública: estudo de caso sobre a CAMEDIS (DF) e o SUS Mediado (RN). Brasília: Faculdade de Administração, Contabilidade, Economia e Gestão de Políticas Públicas da Universidade de Brasília; 2017.

63. Imprensa da DPE/RN. SUS Mediado registra 26% de resolutividade em 2018 e celebra retorno da Secretaria Municipal de Saúde. Natal: Defensoria Pública do Estado do Rio Grande do Norte; 2019 [cited 2019 abril 6]. Available

from: https://www.defensoria.rn.def.br/noticia/sus-mediado-registra-26-de-resolutividade-em-2018-e-celebra-retorno-da-secretaria-municipal.

64. Farias DR, Silva MVC, Santos ASS, Perez KN. Os desafios e as estratégias do poder judiciário no tocantins para a minimização da judicialização da saúde. Revista ESMAT. 2016;8(11):27.

65. Defensoria Pública do Estado do Rio Grande do Sul. A defensoria. Porto Alegre: Defensoria Pública do Estado do Rio Grande do Sul; 2019 [cited 2019 abril 6]. Available from: http://www.defensoria.rs.def.br/apresentacao.

66. Departamento de Informática. Departamento Médico Judiciário. Porto Alegre: Tribunal de Justiça do Estado do Rio Grande do Sul; 2015 [cited 2019 abril 6]. Available from: https://www.tjrs.jus.br/novo/institucional/estrutura-do-judiciario/tribunal-de-justica/setores/departamento-medico-judiciario/.

67. Tavares LLG, Grynberg C, Mascarenhas R, Palheiro PHDM, Filho HGG, Saraiva MLLB. Câmara de Resolução de Litígios de Saúde (CRLS). XI edição do Prêmio Innovare; 16 de dezembro de 2014. Supremo Tribunal Federal, Brasília. Rio de Janeiro: Instituto Innovare; 2014.

68. Tribunal de Justiça do Estado da Bahia. NAT JUS: Núcleo de Apoio Técnico do Poder Judiciário. Salvador: Tribunal de Justiça do Estado da Bahia; 2017 [cited 2019 abril 2]. Available from: https://www.tjba.jus.br/natjus/.

69. Tribunal de Justiça do Estado da Bahia. Decreto judiciário nº 795, de 30 de agosto de 2017. Dispõe sobre a estrutura e funcionamento do NAT-JUS do Tribunal de Justiça. Salvador: Diário da justiça eletrônico; 2017.

70. Ascom TJBA. Núcleo de apoio técnico do judiciário: NAT-JUS reúne-se na assessoria especial da presidência II. Salvador: Tribunal de Justiça do Estado da Bahia; 2018 [updated 28/06/2018; cited 2019 abril 2]. Available from: http://www5.tjba.jus.br/portal/mais-uma-reuniao-do-nucleo-de-apoio-tecnico-do-judiciario-nat-jusfoi-realizada-na-assessoria-especial-da-presidencia-aep-ii-na-ultima-terca-feira-25-06-18/.

71. Secretaria de Saúde do Estado de São Paulo. Resolução SS-54, de 11 de maio de 2012. Aprova, no âmbito da Pasta, estrutura e funcionamento da Comissão de Farmacologia da Secretaria de Estado da Saúde de São Paulo, e dá outras providências. In: Gabinete do Secretário, editor. São Paulo: Diário Oficial Estado de São Paulo; 2012. p. 15.

72. Triagem farmacêutica no juizado especial da fazenda pública de São Paulo. 10ª edição do Prêmio Innovare; 28 de novembro de 2013. Supremo Tribunal Federal, Brasília. Rio de Janeiro: Instituto Innovare; 2013.

73. Paim P, Marqueto A, Lopes IO. Câmara permanente distrital de mediação em saúde: experiência do Distrito Federal. In: CONASS, editor. Direito à saúde. Para Entender a Gestão do SUS - 2015. 1ª ed. Brasília: CONASS; 2015. p. 113.

74. Conselho Federal de Farmácia. Experiências exitosas de farmacêuticos no SUS. 2016;Ano IV(4):192.

75. Macêdo DF, Ataide JAR, Costa ACS, Souza WAR, Rita LPS. Análise da judicialização do direito à saúde, subfinanciamento do setor e políticas públicas: estudo de caso no Estado de Alagoas. Revista de Administração de Roraima-UFRR. 2015;5(2):25.

76. Secretaria de Estado da Saúde de Alagoas. Portaria n. 110, de 2 de maio de 2013. Institui o Núcleo Interinstitucional de Judicialização da Saúde (NIJUS). Maceió: Diário Oficial do Estado de Alagoas; 2013. p. 1.

77. Henrique MC, Mendonça MRL, Braga EA. NatJus e desjudicialização da saúde. In: Santos AO, Lopes LT, editors. Boas Práticas e Diálogos Institucionais. Coletânea Direito à Saúde. 3. 1ª ed. Brasília: CONASS; 2018. p. 292.

78. Tribunal de Justiça do Estado do Pará. Comitê Executivo de Saúde. Belém: Tribunal de Justiça do Estado do Pará,; 2013 [cited 2019 abril 6]. Available from: http://www.tjpa.jus.br/PortalExterno/institucional/Comite-Executivo-de-Saude/330244-Normativos-NAT-Jus.xhtml.

79. Araújo AFS. Judicialização da saúde em Minas Gerais. Novas estratégias de enfrentamento: a criação do Núcleo de Atendimento à Judicialização da Saúde. Cad Ibero-Amer Dir Sanit. 2014;3(2):7.

80. Macedo MB. A judicialização da saúde pública e o diálogo institucional: os espaços interinstitucionais como garantia fundamental de acesso à saúde. Uberlândia: Universidade Federal de Uberlândia; 2016.

81. Galliez MS. Atuação extrajudicial na Saúde Pública: garantia de acesso e qualidade aos serviços. XII edição do Prêmio Innovare; 1º de dezembro de 2015. Supremo Tribunal Fedral, Brasília. Rio de Janeiro: Instituto Innovare; 2015.

82. Faglioni AF, Castelo FA. A especialização das procuradorias dos estados nas questões relativas ao direito à saúde como instrumento eficaz na defesa dos entes estaduais: a experiência da PGE/PR. In: Santos AO, Lopes LT, editors. Boas Práticas e Diálogos Institucionais. Coletânea Direito à Saúde. 3. 1ª ed. Brasília: CONASS; 2018. p. 292.

83. Secretaria da Saúde do Estado do Amapá. Núcleo de Ouvidoria e Apoio Técnico. Macapá: Secretaria da Saúde do Estado do Amapá; 2017 [cited 2019 abril 2]. Available from: https://saude.portal.ap.gov.br/conteudo/cidadao/nucleo-de-ouvidoria-e-apoio-tecnico.

84. Secretaria de Estado da Saúde do Estado de Alagoas. Núcleo de Saúde da PGE facilita respostas para o Judiciário sobre as demandas dos alagoanos Maceió: Secretaria de Estado da Saúde do Estado de Alagoas; 2015

85. Nunes HEN. A prática da mediação e a (des)judicialização da saúde no Maranhão. São Luís: Universidade CEUMA; 2016.

86. Secretaria de Estado da Saúde do Estado do Maranhão. Governo, Prefeitura e defensorias unem esforços para agilizar acesso à Saúde. São Luís: Secretaria de Estado da Saúde do Estado do Maranhão; 2015 [updated 8 de junho de 2015 cited 2019 abril 3]. Available from: http://www.saude.ma.gov.br/governo-prefeitura-e-defensorias-unem-esforcos-para-agilizar-acesso-a-saude/.

87. Oliveira R. Relatório de gestão 2015-2018: avanços e perspectivas. Vitória: Secretaria de Estado da Saúde do Estado do Espírito Santo; 2018.

88. Mariano CM, Furtado ET, Albuquerque FB, Pereira FHLCS. Diálogos sanitários interinstitucionais e a experiência de implantação do NAT-JUS. Revista de Investigações Constitucionais. 2018:21.

89. Tribunal de Justiça do Estado do Ceará. Direito à Saúde. Fortaleza: Tribunal de Justiça do Estado do Ceará; 2017 [cited 2019 abril 2]. Available from: https://www.tjce.jus.br/saude/.

90. Tribunal de Justiça do Estado do Amapá. TJAP inaugura Núcleo de Apoio Técnico Judiciário para demandas da saúde. Macapá: Tribunal de Justiça do Estado do Amapá; 2016 [updated 18 Novembro 2016; cited 2019 abril 2]. Available from: http://www.tjap.jus.br/portal/publicacoes/noticias/5648-justiça-do-amapá-inaugura-núcleo-de-apoio-técnico-judic….

91. Assessoria de Comunicação Social. NAT-JUS: Juízes do Amapá contam com corpo técnico especializado para emitir decisões sobre processos relativos à saúde. Macapá: Tribunal de Justiça do Estado do Amapá; 2017 [updated 16 Mai 2017; cited 2019 abril 2]. Available from: http://www.tjap.jus.br/portal/publicacoes/noticias/6228-nat-jus-juízes-do-amapá-contam-com-corpo-técnico-especializado-para-emitir-decisões-sobre-processos-relativos-à-saúde.html.

92. Tribunal de Justiça do Estado de Alagoas. Resolução nº 18, de 15 de março de 2016. Institui a Câmara Técnica de Saúde no âmbito do Poder Judiciário do Estado de Alagoas e adota providências correlatas. Maceió: Tribunal de Justiça do Estado de Alagoas; 2016. p. 3.

93. Comitê Estadaual das Demandas da Saúde do Rio Grande do Norte. Comitê da Saúde do RN Natal: Secretaria de Comunicação do Tribunal de Justiça do Estado do Rio Grande do Norte; 2016 [cited 2019 abril 6]. Available from: http://comite.tjrn.jus.br/index.php.

94. Ministério Público do Estado de São Paulo. Saúde Pública. São Paulo: Ministério Público do Estado de São Paulo; 2019 [cited 2019 abril 6]. Available from: http://www.mpsp.mp.br/portal/page/portal/Saude_Publica.

95. Reis V. Mario Scheffer fala sobre a queda da judicialização do SUS paulista. Rio de Janeiro: Abrasco; 2018 [cited 2019 abril 6]. Available from: https://www.abrasco.org.br/site/outras-noticias/sistemas-de-saude/mario-scheffer-fala-sobre-queda-da-judicializacao-do-sus-paulista/33433/.

96. Núcleo de Comunicação Social. Notícia: lançado o programa "Acessa SUS", que facilita acesso da população a medicamentos. São Paulo: Ministério Público do Estado de São Paulo; 2017 [cited 2019 abril 6]. Available from: http://www.mpsp.mp.br/portal/page/portal/noticias/noticia?id_noticia=16584466&id_grupo=118.

97. Bittencourt FR. Sistema PES: Procedimento Extrajudicial de Saúde. 14ª edição do Prêmio Innovare; 5 de dezembro de 2017. Supremo Tribunal Federal, Brasília. Rio de Janeiro: Instituto Innovare; 2017. p. 2.

98. Secretaria-Geral e Departamento de Gestão Estratégica. Relatório Anual 2017. Brasília: Conselho Nacional de Justiça; 2017.

99. Tribunal de Justiça de Mato Grosso do Sul. Comitê Estadual do Judiciário para a Saúde. Núcleo de Apoio Técnico - NAT Jus. Campo Grande: Tribunal de Justiça de Mato Grosso do Sul; 2013 [cited 2019 abril 6]. Available from: http://www.tjms.jus.br/nat/.

100. Secretaria de Estado de Saúde do Mato Grosso do Sul. Resolução n.º 009/SES/MS. Campo Grande, 22 de fevereiro de 2018. Regulamenta o cumprimento de determinações judiciais em saúde de pequeno valor nos termos que especifica. Campo Grande: Diário Oficial do Estado; 2018. p. 1.

101. Tribunal de Justiça do Estado do Espírito Santo, Gabinete da presidência. Ato normativo conjunto Nº 44/2018. Dispõe, no âmbito do Poder Judiciário do Estado do Espírito Santo, sobre a utilização do sistema de intimações eletrônicas da Secretaria Estadual de Saúde – SESA/ES, denominado MJ Online (Mandado Judicial Online). Vitória: Tribunal de Justiça do Estado do Espírito Santo; 2018. p. 3.

102. Secretaria de Estado da Saúde do Espírito Santo. Sesa lança Mandado Judicial On-line em parceria com o TJES. Vitória: Secretaria de estado da Saúde do Espírito Santo; 2018 [updated 18/12/2018; cited 2019 abril 3]. Available from: https://saude.es.gov.br/Notícia/sesa-lanca-mandado-judicial-on-line-em-parceria-com-o-tjes
