# Supplementary material for: Strategies Implemented by Public Institutions to Approach the Judicialization of Health Care in Brazil: A Systematic Scoping Review
Source: Front Pharmacol. 2020 Jul 30;11:1128. doi: 10.3389/fphar.2020.01128 (PMC7406659; doi:10.3389/fphar.2020.01128)
Supplement: Supplementary file 4 [file DataSheet_4.pdf]

Supplementary Material

Appendix 4. Characteristics of documents included in the scoping review.

Author	Year of publication	Type of publication
Associação Brasileira de Saúde Coletiva[1]	2018	institutional website
Advocacia Geral da União[2]	2009	institutional website
Araújo[3]	2014	indexed journal article
Asensi & Pinheiro[4]	2015	book
Assis[5]	2015	book section
Bahia *et al.*[6]	2005	book section
Bandeira *et al.*[7]	2019	conference proceedings
Barros[8]	2016	thesis
Bittencourt[9]	2017	conference proceedings
Boldrin[10]	2014	thesis
Casal *et al.*[11]	2009	conference proceedings
Castro[12]	2012	thesis
CEMAS[13]	2019	institutional website
Chayamiti[14]	2018	institutional document
Comitê Estadual das Demandas da Saúde do Rio Grande do Norte[15]	2016	institutional website
Comitê Executivo do Fórum Nacional do Judiciário para Saúde do Conselho Nacional de Justiça[16]	2015	institutional document
Conselho Federal de Farmácia[17]	2013	conference proceedings
Conselho Federal de Farmácia[18]	2016	conference proceedings
Conselho Nacional de Justiça[19]	2015	institutional website
Conselho Nacional de Justiça[20]	2017	institutional document
Conselho Regional de Farmácia do Estado de Minas Gerais[21]	2016	institutional website
Conti[22]	2013	thesis
Costa[23]	2019	conference proceedings
Costa[24]	2014	thesis
Costa[25]	2012	indexed journal article
Cotrim[26]	2017	thesis
Defensoria Pública do Estado do Rio Grande do Sul[27]	2018	institutional document
Diniz[28]	2015	thesis
Duarte[29]	2017	thesis
Faglioni & Castelo[30]	2018	book section
Farias[31]	2016	thesis
Farias *et al.*[32]	2016	indexed journal article
Galliez[33]	2015	conference proceedings
Garofalo *et al.*[34]	2012	conference proceedings
Governo do Estado do Espírito Santo[35]	2007	legislation
Guimarães & Palheiro[36]	2015	book section
Henrique *et al.* [37]	2013	indexed journal article
Henrique *et al.* [38]	2018	book section
Defensoria Pública Estadual do Rio Grande do Norte[39]	2019	institutional website
Júnior [40]	2017	thesis
Junior [41]	2008	thesis
Macêdo *et al.* [42]	2015	indexed journal article
Macedo [43]	2016	thesis
Mariano *et al.*[44]	2018	indexed journal article
Mauad *et al.*[45]	2009	conference proceedings
Ministério da Saúde[46]	2013	institutional website
Ministério Público do Estado de São Paulo[47]	2017	institutional website
Ministério Público do Estado de São Paulo[48]	2019	institutional website

Author	Year of publication	Type of publication
Naffah-Filho *et al.*[49]	2010	non-indexed journal article
Nantes & Dobashi [50]	2015	book section
Nunes [51]	2016	thesis
Oliveira [52]	2018	institutional document
Orsatto [53]	2014	conference proceedings
Paim *et al.*[54]	2015	book section
Pereira [55]	2012	thesis
Pereira & Carneiro[56]	2012	conference proceedings
Perin *et al.* [57]	2008	conference proceedings
Pinheiro [58]	2010	conference proceedings
Pontarolli *et al.* [59]	2015	book section
Prefeitura Municipal de Lages [60]	2015	institutional website
Queiroz [61]	2013	thesis
Reis [62]	2011	conference proceedings
Ringeisen [63]	2016	thesis
Sanchez LA *et al.* [64]	2008	conference proceedings
Sant'Ana [65]	2017	thesis
Schulze [66]	2018	book section
Secretaria da Saúde do Estado do Amapá [67]	2017	institutional website
Secretaria de Estado da Saúde do Espírito Santo[68]	2018	institutional website
Secretaria de Estado da Saúde do Estado de Alagoas[69]	2013	legislation
Secretaria de Estado da Saúde do Estado de Alagoas[70]	2015	institutional website
Secretaria de Estado da Saúde do Estado de São Paulo[71]	2019	institutional website
Secretaria de Estado da Saúde do Estado do Maranhão[72]	2015	institutional website
Secretaria de Estado da Saúde do Paraná[73]	2008	institutional document
Secretaria de Estado de Saúde do Mato Grosso do Sul[74]	2018	legislation
Secretaria de Saúde do Estado de São Paulo[75]	2012	legislation
Secretaria de Saúde do Município de Ribeirão Preto[76]	2017	institutional website
Unknown author[77]	2013	conference proceedings
Silva[78]	2012	thesis
Simabuku *et al.*[79]	2015	indexed journal article
Simões[80]	2015	thesis
Siqueira *et al.* [81]	2018	book section
Souza[82]	2016	thesis
Tavares *et al.*[83]	2010	conference proceedings
Tavares *et al.*[84]	2009	conference proceedings
Tavares *et al.*[85]	2014	conference proceedings
Teixeira [86]	2011	thesis
Toma *et al.*[87]	2015	book
Toma *et al.*[88]	2017	indexed journal article
Tribunal de Justiça da Bahia[89]	2018	institutional website
Tribunal de Justiça de Mato Grosso do Sul[90]	2013	institutional website
Tribunal de Justiça do Amapá[91]	2016	institutional website
Tribunal de Justiça do Estado da Bahia[92]	2017	legislation
Tribunal de Justiça do Estado da Bahia[93]	2017	institutional website
Tribunal de Justiça do Estado de Alagoas[94]	2016	legislation
Tribunal de Justiça do Estado do Amapá[95]	2017	institutional website
Tribunal de Justiça do Estado do Ceará[96]	2017	institutional website
Tribunal de Justiça do Estado do Espírito Santo[97]	2018	legislation
Tribunal de Justiça do Estado do Pará[98]	2013	institutional website
Tribunal de Justiça do Estado do Rio Grande do Sul[99]	2015	institutional website
Tribunal de Justiça do Maranhão[100]	2018	legislation
Ungaro[101]	2011	conference proceedings
Yoshinaga[102]	2011	non-indexed journal article

References

1. Reis V. Mario Scheffer fala sobre a queda da judicialização do SUS paulista. Rio de Janeiro: Abrasco; 2018 [cited 2019 abril 6]. Available from: https://www.abrasco.org.br/site/outras-noticias/sistemas-de-saude/mario-scheffer-fala-sobre-queda-da-judicializacao-do-sus-paulista/33433/.
2. Advocacia Geral da União. CIRADS. Brasília: Advocacia-Geral da União; 2009 [updated 2013; cited 2019 abril 6]. Available from: http://www.agu.gov.br/page/content/detail/id_conteudo/97046.
3. Araújo AFS. Judicialização da saúde em Minas Gerais. Novas estratégias de enfrentamento: a criação do Núcleo de Atendimento à Judicialização da Saúde. Cad Ibero-Amer Dir Sanit. 2014;3(2):7.
4. Asensi FD, Pinheiro R, Coordenadores. Judicialização da saúde no Brasil: dados e experiência. Brasília: Conselho Nacional de Justiça; 2015. 142 p.
5. Assis G. Mediação sanitária: direito, saúde e cidadania. In: CONASS, editor. Direito à saúde. Para Entender a Gestão do SUS - 2015. 1ª ed. Brasília: CONASS; 2015. p. 113.
6. Bahia GOM, Lamb L, Nicoletti RHA, Cataneli RCB, Luiz VR. A política de medicamentos no Estado do Paraná. In: Saúde. CONASS, editor. Relatório final do I encontro do CONASS para troca de experiências. CONASS Documenta. Brasília: CONASS; 2005. p. 140.
7. Bandeira LM, Bandeira RC, Costa CA, Santos JBFMS, Silva HA, Furtado EAV. A atuação do Núcleo de Apoio à Procuradoria Adjunta de Suporte à Saúde no município de Três Rios como um norte para a contenção saudável da judicialização em saúde. Rio de Janeiro: IdeiaSUS - Banco de Práticas e Soluções em Saúde e Ambiente; 2019 [cited 20191 abril 6]. Available from: http://www.ideiasus.fiocruz.br/portal/index.php/banco-de-praticas.
8. Barros LD. Judicialização do direito à saúde: uma análise acerca do fornecimento judicial de medicamentos no Estado de Pernambuco. Recife: Universidade Federal de Pernambuco; 2016.
9. Bittencourt FR. Sistema PES: Procedimento Extrajudicial de Saúde. 14ª edição do Prêmio Innovare; 5 de dezembro de 2017. Supremo Tribunal Federal, Brasília. Rio de Janeiro: Instituto Innovare; 2017. p. 2.
10. Boldrin PHM. A efetividade da Comissão de Análise de Solicitações Especiais da judicialização das políticas de saúde referentes às demandas ajuizadas pela Defensoria Pública do Estado de São Paulo - Regional Ribeirão Preto. Ribeirão Preto: Universidade de São Paulo; 2014.
11. Casal VAS, Motta LJ, Carvalho A, Maximiano VAZ. Obtenção de medicamentos extrajudicialmente. VI Prêmio Innovare: Justiça Rápida e Eficaz; 17 de dezembro de 2009. Supremo Tribunal Federal, Brasília. Rio de Janeiro: Instituto Innovare; 2009.
12. Castro KRTR. Os juízes diante da judicialização da saúde: o NAT como instrumento de aperfeiçoamento das decisões judiciais na área da saúde. Rio de Janeiro: Fundação Getúlio Vargas; 2012.
13. CEMAS. Comitê Executivo para Monitoramento das Ações da Saúde no Estado do Tocantins – CEMAS. Palmas: Tribunal de Justiça do Estado do Tocantins; 2019 [cited 2019 abril 6]. Available from: http://wwa.tjto.jus.br/saude/.
14. Chayamiti EMPC, Coordenadora. Manual do serviço de atenção domiciliar. 2ª revisão ed. Ribeirão Preto: Secretaria municipal da saúde de Ribeirão Preto; 2018. 64 p.
15. Comitê Estadual das Demandas da Saúde do Rio Grande do Norte. Comitê da Saúde do RN Natal: Secretaria de Comunicação do Tribunal de Justiça do Estado do Rio Grande do Norte; 2016 [cited 2019 abril 6]. Available from: http://comite.tjrn.jus.br/index.php.
16. Comitê Executivo do Fórum Nacional do Judiciário para Saúde do Conselho Nacional de Justiça. Ação de planejamento e de gestão sistêmicos com foco na saúde. Porto Alegre: Comitê Executivo do Rio Grande do Sul; 2015. 287 p.
17. Conselho Federal de Farmácia. Experiências exitosas de farmacêuticos no SUS. 2013;ano I(1):82.
18. Conselho Federal de Farmácia. Experiências exitosas de farmacêuticos no SUS. 2016;Ano IV(4):192.

19. Vasconcellos J. Núcleo de conciliação de Lages/SC soluciona 90% dos conflitos na saúde. Brasília: CNJ; 2015 [updated 08/05/2015; cited 2019 abril 3]. Available from: http://cnj.jus.br/noticias/cnj/79305-nucleo-de-conciliacao-de-lages-sc-soluciona-90-dos-conflitos-na-saude.

20. Secretaria-Geral e Departamento de Gestão Estratégica. Relatório Anual 2017. Brasília: Conselho Nacional de Justiça; 2017.

21. Secretaria Estadual de Saúde. SES-MG e Defensoria Pública estabelecem parceria pioneira para agilizar assistência farmacêutica no Norte de Minas. Belo Horizonte: Conselho Regional de Farmácia do Estado de Minas Gerais; 2016 [updated 08/03/2016; cited 2019 abril 6]. Available from: http://www.crfmg.org.br/site/Noticias/SES-MG-e-Defensoria-Publica-estabelecem-parceria-pioneira-para-agilizar-assistencia-farmaceutica-no-Norte-de-Minas.

22. Conti MA. Avaliação das demandas judiciais por acesso a medicamentos no Distrito Federal. Brasília: Universidade de Brasília. Faculdade de Ciências da Saúde; 2013.

23. Costa CA. Gestão de pessoal e educação permanente em saúde pública como viés para redução do ativismo judicial. Rio de Janeiro: IdeiaSUS - Banco de Práticas e Soluções em Saúde e Ambiente; 2019 [cited 2019 abril 6]. Available from: http://www.ideiasus.fiocruz.br/portal/index.php/banco-de-praticas/busca-avancada.

24. Costa IC. Judicialização da saúde e a Câmara Técnica de Saúde no tribunal de justiça da Paraíba. Guabira: Centro de Humanidades da Universidade Estadual da Paraíba; 2014.

25. Costa KS, Nascimento-Jr JM. HÓRUS: inovação tecnológica na assistência farmacêutica no sistema único de saúde. Revista de Saúde Pública. 2012;46:91-9

26. Cotrim TP. Mediação como instrumento de ação pública: estudo de caso sobre a CAMEDIS (DF) e o SUS Mediado (RN). Brasília: Faculdade de Administração, Contabilidade, Economia e Gestão de Políticas Públicas da Universidade de Brasília; 2017.

27. Defensoria Pública do Estado do Rio Grande do Sul. *Relatório anual 2018: dados, ações, projetos e números.* Porto Alegre: Defensoria Pública do Estado do Rio Grande do Sul; 2018.

28. Diniz IM. Direito à saúde e judicialização: uma análise da atuação do Conselho Nacional de Justiça no aprimoramento da prestação jurisdicional nas demandas de saúde. São Luís: Universidade Federal do Maranhão; 2015.

29. Duarte VG. Arranjos e diálogos institucionais para enfrentamento da judicialização da saúde: uma análise dos modelos de assessoramento técnico (NAT's). Limeira: Universidade Estadual de Campinas; 2017.

30. Faglioni AF, Castelo FA. A especialização das procuradorias dos estados nas questões relativas ao direito à saúde como instrumento eficaz na defesa dos entes estaduais: a experiência da PGE/PR. In: Santos AO, Lopes LT, editors. Boas Práticas e Diálogos Institucionais. Coletânea Direito à Saúde. 3. 1ª ed. Brasília: CONASS; 2018. p. 292.

31. Farias DR. Judicialização da saúde: aspectos processuais e institucionais na efetivação do direito à saúde pública no Estado do Tocantins. Palmas: Universidade Federal do Tocantins; 2016.

32. Farias DR, Silva MVC, Santos ASS, Perez KN. Os desafios e as estratégias do poder judiciário no tocantins para a minimização da judicialização da saúde. Revista ESMAT. 2016;8(11):27.

33. Galliez MS. Atuação extrajudicial na Saúde Pública: garantia de acesso e qualidade aos serviços. XII edição do Prêmio Innovare; 1º de dezembro de 2015. Supremo Tribunal Fedral, Brasília. Rio de Janeiro: Instituto Innovare; 2015.

34. Garofalo G, Baseio C, Campos L, Alonso MS. Atenção farmacêutica na diminuição da prescrição e do uso irracional de medicamentos na judicialização do SUS. Prêmio Nacional de Incentivo à Promoção do Uso Racional de Medicamentos – 2011/Ministério da Saúde, Secretaria de Ciência, Tecnologia e Insumos Estratégicos, Departamento de Ciência e Tecnologia. Brasília: Ministério da Saúde; 2012. p. 102.

35. Governo do Estado do Espírito Santo. Decreto n° 1956-R, de 07 de novembro de 2007: Aprova a Política Farmacêutica do Estado do Espírito Santo e dá outras providências. Vitória: Gabinete do governador; 2007. p. 5.

36. Guimarães RCM, Palheiro PHD. Medidas adotadas para enfrentar a judicialização na secretaria de saúde do Estado do Rio De Janeiro e a experiência da Câmara de Resolução de Litígios de Saúde. In: CONASS, editor. Direito à saúde. Para Entender a Gestão do SUS – 2015. 1ª ed. Brasília: CONASS; 2015. p. 113.

37. Henrique MC, Brito JOB, Mel MDSM. Eficiência na solução das demandas de judicialização da saúde na Comarca de Araguaina-TO. Cad IberAmer Direito Sanit. 2013;2(2):19.

38. Henrique MC, Mendonça MRL, Braga EA. NatJus e desjudicialização da saúde. In: Santos AO, Lopes LT, editors. Boas Práticas e Diálogos Institucionais. Coletânea Direito à Saúde. 3. 1ª ed. Brasília: CONASS; 2018. p. 292.

39. Imprensa da DPE/RN. SUS Mediado registra 26% de resolutividade em 2018 e celebra retorno da Secretaria Municipal de Saúde. Natal: Defensoria Pública do Estado do Rio Grande do Norte; 2019 [cited 2019 abril 6]. Available from: https://www.defensoria.rn.def.br/noticia/sus-mediado-registra-26-de-resolutividade-em-2018-e-celebra-retorno-da-secretaria-municipal.

40. Júnior LMRR. A mediação como instrumento de efetivação do direito à saúde: análise dos relatórios de antendimentos realizados pelo programa "SUS Mediado", no ano de 2014. Recife: Universidade Católica de Pernambuco; 2017.

41. Junior PBR. A judicialização do acesso a medicamentos: A perspectiva da secretaria municipal de saúde do Rio de Janeiro. Rio de Janeiro: Fundação Getulio Vargas - Escola Brasileira de Administração Pública e de Empresas; 2008.

42. Macêdo DF, Ataide JAR, Costa ACS, Souza WAR, Rita LPS. Análise da judicialização do direito à saúde, subfinanciamento do setor e políticas públicas: estudo de caso no Estado de Alagoas. Revista de Administração de Roraima-UFRR. 2015;5(2):25.

43. Macedo MB. A judicialização da saúde pública e o diálogo institucional: os espaços interinstitucionais como garantia fundamental de acesso à saúde. Uberlândia: Universidade Federal de Uberlândia; 2016.

44. Mariano CM, Furtado ET, Albuquerque FB, Pereira FHLCS. Diálogos sanitários interinstitucionais e a experiência de implantação do NAT-JUS. Revista de Investigações Constitucionais. 2018:21.

45. Mauad MLME, Tanaka NYY, Fazan WC. Protocolo inicial da comissão de análise de solicitações especiais para dieta enteral. In: Coordenação-Geral da Política de Alimentação e Nutrição, editor. II Mostra de Alimentação e Nutrição do SUS I Seminário Internacional de Nutrição na Atenção Primária: trabalhos selecionados para apresentação oral. Comunicação e Educação em Saúd. Brasília: Ministério da Saúde; 2009. p. 122.

46. Ministério da Saúde, Assistência Farmacêutica. Sistema Hórus. Brasília: Ministério da Saúde; 2013 [cited 2019 abril 2]. Available from: http://portalms.saude.gov.br/assistencia-farmaceutica/sistema-horus/sobre-o-sistema.

47. Núcleo de Comunicação Social. Notícia: lançado o programa "Acessa SUS", que facilita acesso da população a medicamentos. São Paulo: Ministério Público do Estado de São Paulo; 2017 [cited 2019 abril 6]. Available from: http://www.mpsp.mp.br/portal/page/portal/noticias/noticia?id_noticia=16584466&id_grupo=118

48. Ministério Público do Estado de São Paulo. Saúde Pública. São Paulo: Ministério Público do Estado de São Paulo; 2019 [cited 2019 abril 6]. Available from: http://www.mpsp.mp.br/portal/page/portal/Saude_Publica.

49. Naffah-Filho M, Chieffi AL, Correa MCMMA. S-Codes: um novo sistema de informações sobre ações judiciais da Secretaria de Estado da Saúde de São Paulo. BEPA. 2010;7(84):12.

50. Nantes LFL, Dobashi BF. A experiência da secretaria estadual de saúde de Mato Grosso do Sul no enfrentamento das ações judiciais em saúde. In: CONASS, editor. Direito à saúde. Para Entender a Gestão do SUS - 2015. 1ª ed. Brasileiro: CONASS; 2015. p. 113.

51. Nunes HEN. A prática da mediação e a (des)judicialização da saúde no Maranhão. São Luís: Universidade CEUMA; 2016.

52. Oliveira R. Relatório de gestão 2015-2018: avanços e perspectivas. Vitória: Secretaria de Estado da Saúde do Estado do Espírito Santo; 2018.

53. Orsatto SD. Núcleo de conciliação de demandas em saúde. XI edição do Prêmio Innovare; 16 de dezembro de 2014. Supremo Tribunal Federal, Brasília. Rio de Janeiro: Instituto Innovare; 2014.

54. Paim P, Marqueto A, Lopes IO. Câmara permanente distrital de mediação em saúde: experiência do Distrito Federal. In: CONASS, editor. Direito à saúde. Para Entender a Gestão do SUS - 2015. 1ª ed. Brasília: CONASS; 2015. p. 113.

55. Pereira LR. Judicialização da saúde e mudanças organizacionais: o impacto das decisões judiciais na estrutura da Secretaria Estadual de Saúde de Minas Gerais (SES-MG). Belo Horizonte: Escola de Governo Professor Paulo Neves de Carvalho; 2012.

56. Pereira LR, Carneiro R. Judicialização da saúde e mudanças organizacionais: o impacto das decisões judiciais na estrutura da secretaria de Estado de saúde de Minas Gerais (SES-MG). V Encontro da Divisão de Administração Pública/APB da ANPAD - EnAPG; 18 a 20 de novembro de 2012, Salvador/BA Maringá: Associação Nacional de Pós-Graduação e Pesquisa em Administração; 2012.

57. Perin IG, Tassinari MTR, Santos MG, Santos IES, Larogian APA, Teixeira VM, et al. Desafios e avanços da comissão de análise de solicitações especiais visando a equidade e o aprimoramento da gestão do SUS. In: Unidade de Desenvolvimento e Melhoria das Organizações, editor. Prêmio Mario Covas, 5ª edição, ciclo 2008; Theatro Municipal de São Paulo. São Paulo: Secretaria Estadual de Planejamento e Gestão; 2008.

58. Pinheiro TP. Comitê Interinstitucional de Resolução Administrativa de Demandas da Saúde (CIRADS). VII edição do Prêmio Innovare: Justiça sem burocracia e acesso do preso à Justiça; 3 de dezembro de 2010. Tribunal Federal de Justiça, Brasília. Rio de Janeiro: Instituto Innovare; 2010.

59. Pontarolli DRS, Silva GRPP, Strapasson GC. O enfrentamento das demandas judiciais por medicamentos na secretaria de Estado da saúde do Paraná. In: CONASS, editor. Direito à saúde. Para Entender a Gestão do SUS - 2015. 1ª ed. Brasília: CONASS; 2015. p. 113.

60. Prefeitura Municipal de Lages. Núcleo de Medicamentos. Município e Tribunal de Justiça firmarão convênio. Lages: Prefeitura de Lages; 2015 [updated 23/03/2015; cited 2019 abril 3]. Available from: http://www.lages.sc.gov.br/noticia/5800/municipio-e-tribunal-de-justica-firmarao-convenio/.

61. Queiroz CC. A mediação como instrumento concretizador do direito fundamental à saúde: uma alternativa à judicialização de conflitos. Natal: Universidade Federal do Rio Grande do Norte; 2013.

62. Reis BB. Defensoria especializada da saúde. In: Associação Nacional de Defensores Públicos, editor. Concurso de práticas exitosas 2011 X Congresso Nacional de Defensores Públicos (ANADEP); 15 a 18 de novembro, Centro de Convenções de Natal -RN. Brasília: ANADEP; 2011. p. 16.

63. Ringeisen ATS. Mediação de conflitos no sistema único de saúde: visões e práticas de uma experiência no município de Natal/RN. Natal: Universidade Federal do Rio Grande do Norte; 2016.

64. Sanchez LA, Santos LN, Gomes RAC, Chieffi AL, Souza CZB, Camargo CR, et al. Uma parceria inovadora para fazer frente ao fenômeno da "judicialização das políticas públicas de saúde": a otimização da gestão pública. In: Unidade de Desenvolvimento e Melhoria das Organizações, editor. Prêmio Mario Covas, 5ª edição, ciclo 2008; Theatro Municipal de São Paulo. São Paulo: Secretaria Estadual de Planejamento e Gestão; 2008.

65. Sant'Ana RN. A judicialização como instrumento de acesso à saúde: propostas de enfretamento da injustiça na saúde pública. Brasília: Centro Universitário de Brasília; 2017.

66. Schulze M. A desjudicialização da saúde no Rio Grande do Sul: cabal redução do número das ações ativas e estratégia de interiorização. In: Santos AO, Lopes LT, editors. Boas Práticas e Diálogos Institucionais. Coletânea Direito à Saúde. 3. 1ª ed. Brasília: CONASS; 2018. p. 292.

67. Secretaria da Saúde do Estado do Amapá. Núcleo de Ouvidoria e Apoio Técnico. Macapá: Secretaria da Saúde do Estado do Amapá; 2017 [cited 2019 abril 2]. Available from: https://saude.portal.ap.gov.br/conteudo/cidadao/nucleo-de-ouvidoria-e-apoio-tecnico.

68. Secretaria de Estado da Saúde do Espírito Santo. Sesa lança Mandado Judicial On-line em parceria com o TJES. Vitória: Secretaria de estado da Saúde do Espírito Santo; 2018 [updated 18/12/2018; cited 2019 abril 3]. Available from: https://saude.es.gov.br/Notícia/sesa-lanca-mandado-judicial-on-line-em-parceria-com-o-tjes.

69. Secretaria de Estado da Saúde de Alagoas. Portaria n. 110, de 2 de maio de 2013. Institui o Núcleo Interinstitucional de Judicialização da Saúde (NIJUS). Maceió: Diário Oficial do Estado de Alagoas; 2013. p. 1.

70. Secretaria de Estado da Saúde do Estado de Alagoas. Núcleo de Saúde da PGE facilita respostas para o Judiciário sobre as demandas dos alagoanos Maceió: Secretaria de Estado da Saúde do Estado de Alagoas; 2015.

71. Secretaria de Estado da Saúde do Estado de São Paulo. Comissão de Farmacologia. São Paulo: Secretaria de Estado da Saúde; 2019 [cited 2019 abril 6]. Available from: http://saude.sp.gov.br/ses/perfil/gestor/comissao-de-farmacologia/.

72. Secretaria de Estado da Saúde do Estado do Maranhão. Governo, Prefeitura e defensorias unem esforços para agilizar acesso à Saúde. São Luís: Secretaria de Estado da Saúde do Estado do Maranhão; 2015 [updated 8 de junho de 2015 cited 2019 abril 3]. Available from: http://www.saude.ma.gov.br/governo-prefeitura-e-defensorias-unem-esforcos-para-agilizar-acesso-a-saude/.

73. Secretaria de Estado da Saúde do Paraná, Superintendência de Gestão de Sistemas de Saúde. Assistência Farmacêutica. Curitiba: Secretaria de Estado da Saúde do Paraná; 2008. p. 36.

74. Secretaria de Estado de Saúde do Mato Grosso do Sul. Resolução n.º 009/SES/MS. Campo Grande, 22 de fevereiro de 2018. Regulamenta o cumprimento de determinações judiciais em saúde de pequeno valor nos termos que especifica. Campo Grande: Diário Oficial do Estado; 2018. p. 1.

75. Secretaria de Saúde do Estado de São Paulo. Resolução SS-54, de 11 de maio de 2012. Aprova, no âmbito da Pasta, estrutura e funcionamento da Comissão de Farmacologia da Secretaria de Estado da Saúde de São Paulo, e dá outras providências. In: Gabinete do Secretário, editor. São Paulo: Diário Oficial Estado de São Paulo; 2012. p. 15.

76. Secretaria da Saúde do Município de Ribeirão Preto. Serviço de Atenção Domiciliar – SAD. Ribeirão Preto: Prefeitura da cidade de Ribeirão Preto; 2017 [cited 2019 abril 6]. Available from: https://www.ribeiraopreto.sp.gov.br/ssaude/programas/sad/i16indice.php.

77. Triagem farmacêutica no juizado especial da fazenda pública de São Paulo. 10ª edição do Prêmio Innovare; 28 de novembro de 2013. Supremo Tribunal Federal, Brasília. Rio de Janeiro: Instituto Innovare; 2013.

78. Silva MV. O processo decisório judicial e a assessoria técnica: a argumentação jurídica e médico-sanitária na garantia do direito à assistência terapêutica no Sistema Único de Saúde. Rio de Janeiro: Fundação Oswaldo Cruz; 2012.

79. Simabuku EMG, Catanheide ID, Biella CA, Rabelo RB, Santos VCC, Petramale CA. Comissão nacional de incorporação de tecnologias no SUS e a judicialização do acesso à saúde. Revista Eletrônica Gestão & Saúde. 2015;6(Supl. 4):18.

80. Simões FHS. Judicialização do acesso ao tratamento do diabetes no Estado de Minas Gerais. Belo Horizonte: Universidade Federal de Minas Gerais; 2015.

81. Siqueira PSF, Martins MA, Domingues R. S-CODES: Sistema informatizado para o trato da judicialização. In: Santos AO, Lopes LT, editors. Boas Práticas e Diálogos Institucionais. Coletânea Direito à Saúde. 3. 1ª ed. Brasília: CONASS; 2018. p. 292.

82. Souza AM. A atuação em rede de instituições governamentais na resolução de conflitos sobre demandas sanitárias no Rio de Janeiro. Rio de Janeiro: Fundação Getúlio Vargas; 2016.

83. Tavares GRP, Silva DM, Barcelos PC, Ribeiro C, Moreira GL. Diagnóstico das ações judiciais direcionadas à secretaria de Estado da saúde do Espírito Santo. In: Conselho Nacional de Secretários de Estado da Administração, editor. III Congresso Consad de Gestão Pública; 15 a 17 de março de 2010. Centro de Convenções Ulisses Guimarães, Brasília/DF. Brasília: Consad; 2010. p. 31

84. Tavares GRP, Silva DM, Bernardos A. Sistema de regulação da dispensação de medicamentos excepcionais. In: Conselho Nacional de Secretários de Estado da Administração, editor. II Congresso Consad de Gestão Pública; 6 a 8 de maio de 2009. Centro de Convenções Ulysses Guimarães, Brasília-DF. Brasília: CONSAD; 2009. p. 24.

85. Tavares LLG, Grynberg C, Mascarenhas R, Palheiro PHDM, Filho HGG, Saraiva MLLB. Câmara de Resolução de Litígios de Saúde (CRLS). XI edição do Prêmio Innovare; 16 de dezembro de 2014. Supremo Tribunal Federal, Brasília. Rio de Janeiro: Instituto Innovare; 2014.

86. Teixeira MF. Criando alternativas ao processo de judicialização da saúde: o sistema de pedido administrativo, uma iniciativa pioneira do estado e município do Rio de Janeiro". Rio de Janeiro: Fundação Oswaldo Cruz; 2011.

87. Toma TS, Soares AC, Bortoli MC, Pirotta KCM, Venâncio SI, Derbli M, et al. Avaliação de tecnologias e inovação em saúde no SUS: desafios e propostas para a gestão. São Paulo: Instituto de Saúde; 2015. 344 p.

88. Toma TS, Soares AC, Siqueira PSFd, Domingues R. Estratégias para lidar com as ações judiciais de medicamentos no estado de São Paulo. Cad Ibero-Amer Dir Sanit. 2017;6(1):19.

89. Ascom TJBA. Núcleo de apoio técnico do judiciário: NAT-JUS reúne-se na assessoria especial da presidência II. Salvador: Tribunal de Justiça do Estado da Bahia; 2018 [updated 28/06/2018; cited 2019 abril 2]. Available from: http://www5.tjba.jus.br/portal/mais-uma-reuniao-do-nucleo-de-apoio-tecnico-do-judiciario-nat-jusfoi-realizada-na-assessoria-especial-da-presidencia-aep-ii-na-ultima-terca-feira-25-06-18/.

90. Tribunal de Justiça de Mato Grosso do Sul. Comitê Estadual do Judiciário para a Saúde. Núcleo de Apoio Técnico - NAT Jus. Campo Grande: Tribunal de Justiça de Mato Grosso do Sul; 2013 [cited 2019 abril 6]. Available from: http://www.tjms.jus.br/nat/.

91. Tribunal de Justiça do Estado do Amapá. TJAP inaugura Núcleo de Apoio Técnico Judiciário para demandas da saúde. Macapá: Tribunal de Justiça do Estado do Amapá; 2016 [updated 18 Novembro 2016; cited 2019 abril 2]. Available from: http://www.tjap.jus.br/portal/publicacoes/noticias/5648-justiça-do-amapá-inaugura-núcleo-de-apoio-técnico-judic….

92. Tribunal de Justiça do Estado da Bahia. Decreto judiciário nº 795, de 30 de agosto de 2017. Dispõe sobre a estrutura e funcionamento do NAT-JUS do Tribunal de Justiça. Salvador: Diário da justiça eletrônico; 2017.

93. Tribunal de Justiça do Estado da Bahia. NAT JUS: Núcleo de Apoio Técnico do Poder Judiciário. Salvador: Tribunal de Justiça do Estado da Bahia; 2017 [cited 2019 abril 2]. Available from: https://www.tjba.jus.br/natjus/.

94. Tribunal de Justiça do Estado de Alagoas. Resolução nº 18, de 15 de março de 2016. Institui a Câmara Técnica de Saúde no âmbito do Poder Judiciário do Estado de Alagoas e adota providências correlatas. Maceió: Tribunal de Justiça do Estado de Alagoas; 2016. p. 3.

95. Assessoria de Comunicação Social. NAT-JUS: Juízes do Amapá contam com corpo técnico especializado para emitir decisões sobre processos relativos à saúde. Macapá: Tribunal de Justiça do Estado do Amapá; 2017 [updated 16 Mai 2017; cited 2019 abril 2]. Available from: http://www.tjap.jus.br/portal/publicacoes/noticias/6228-nat-jus-juízes-do-amapá-contam-com-corpo-técnico-especializado-para-emitir-decisões-sobre-processos-relativos-à-saúde.html.

96. Tribunal de Justiça do Estado do Ceará. Direito à Saúde. Fortaleza: Tribunal de Justiça do Estado do Ceará; 2017 [cited 2019 abril 2]. Available from: https://www.tjce.jus.br/saude/.

97. Tribunal de Justiça do Estado do Espírito Santo, Gabinete da presidência. Ato normativo conjunto Nº 44/2018. Dispõe, no âmbito do Poder Judiciário do Estado do Espírito Santo, sobre a utilização do sistema de intimações eletrônicas da Secretaria Estadual de Saúde – SESA/ES, denominado MJ Online (Mandado Judicial Online). Vitória: Tribunal de Justiça do Estado do Espírito Santo; 2018. p. 3.

98. Tribunal de Justiça do Estado do Pará. Comitê Executivo de Saúde. Belém: Tribunal de Justiça do Estado do Pará,; 2013 [cited 2019 abril 6]. Available from: http://www.tjpa.jus.br/PortalExterno/institucional/Comite-Executivo-de-Saude/330244-Normativos-NAT-Jus.xhtml.

99. Departamento de Informática. Departamento Médico Judiciário. Porto Alegre: Tribunal de Justiça do Estado do Rio Grande do Sul; 2015 [cited 2019 abril 6]. Available from: https://www.tjrs.jus.br/novo/institucional/estrutura-do-judiciario/tribunal-de-justica/setores/departamento-medico-judiciario/.

100. Tribunal de Justiça do Maranhão. Portaria-GP nº 683/2018. Dispõe sobre a composição da Comissão do Comitê Estadual de Saúde do Estado do Maranhão. São Luís: Tribunal de Justiça do Maranhão; 2018. p. 1.

101. Ungaro G. O combate às fraudes judiciais em ações para fornecimento de medicamentos no Estado de São Paulo. VIII edição do Prêmio Innovare; 15 de dezembro de 2011. Tribunal Federal de Justiça, Brasília. Rio de Janeiro; 2011.

102. Yoshinaga JY. Judicialização do direito à saúde: a experiência do estado de São Paulo na adoção de estratégias judiciais e extrajudiciais para lidar com esta realidade. Salvador: Revista Eletrônica sobre a Reforma do Estado; 2011 [cited 2019 abril 6]. [Available from: http://www.direitodoestado.com.br/rere/edicao/24.
